# Structural Characterization and Immunomodulatory Activity of an Acidic Polysaccharide from *Rhodomyrtus tomentosa* (Aiton) Hassk. Fruits

**DOI:** 10.3390/molecules31081365

**Published:** 2026-04-21

**Authors:** Huihui Yin, Guoqing Yan, Yunfu Huang, Xueyan Zeng, Shenhong He, Tianyan Lan, Wei Liu

**Affiliations:** 1 Guangxi Key Laboratory of Veterinary Biotechnology, Guangxi Veterinary Research Institute, Nanning 530001, China; huihui981@foxmail.com (H.Y.);; 2 Key Laboratory of China (Guangxi)—ASEAN Cross-Border Animal Disease Prevention and Control, Ministry of Agriculture and Rural Affairs of China, Nanning 530001, China

**Keywords:** *Rhodomyrtus tomentosa* (Aiton) Hassk. fruit, polysaccharide, structural characterization, immunomodulatory activity

## Abstract

A polysaccharide from *Rhodomyrtus tomentosa* (Aiton) Hassk. fruit (RTFP-2b) was isolated and purified. RTFP-2b has a molecular weight of 22.995 kDa and consists of nine monosaccharides, with arabinose (38.68%), galactose (21.86%), and galacturonic acid (14.83%) as its major components. Methylation and NMR analyses revealed dominant glycosidic linkages, including α-L-Ara*f*-(1→, →4)-α-D-Gal*p*A-(1→, →4)-α-D-Gal*p*-(1→, →5)-α-L-Ara*f*-(1→, →3)-α-L-Ara*f*-(1→, →2)-α-L-Rha*p*-(1→, and →3,4,6)-β-D-Gal*p*-(1→. Bioactivity assays using lipopolysaccharide (LPS)-stimulated RAW264.7 cells showed that RTFP-2b exhibits dose-dependent immunomodulatory properties. When administered at lower concentrations (100–200 μg/mL), RTFP-2b enhanced phagocytosis and IL-1β production. At higher concentrations (300–400 μg/mL), it significantly suppressed nitric oxide and showed biphasic regulation of IL-1β, but unexpectedly increased IL-6 levels in LPS-stimulated RAW264.7 cells. These immunomodulatory effects of RTFP-2b at higher doses were accompanied by inhibition of NF-κB signaling. These findings indicate that RTFP-2b is a structurally distinct acidic polysaccharide with dose-dependent immunomodulatory properties, suggesting its potential application in functional foods or pharmaceuticals.

## 1. Introduction

Chronic inflammation is a central pathological mechanism in many diseases, including diabetes, atherosclerosis, cardiovascular disorders, and certain cancers [1,2]. Conventional pharmacological treatments, such as non-steroidal anti-inflammatory drugs and glucocorticoids, frequently induce adverse effects like gastrointestinal injury and immunosuppression with prolonged administration [3,4,5]. This limitation has spurred interest in plant-derived natural compounds, which often exhibit better safety profiles and multi-target regulatory capabilities, including the modulation of immune and inflammatory responses. Polysaccharides, in particular, have garnered significant interest for their marked bioactivity, biocompatibility, and structural diversity, properties that facilitate specific receptor interactions and immune response modulation [6,7]. Acidic polysaccharides have shown particular efficacy in alleviating oxidative stress, cytokine-mediated inflammatory injury and immunomodulatory effect, underscoring their potential for development in functional foods and precision medicine [8,9,10].

*Rhodomyrtus tomentosa* (Aiton) Hassk. (*R. tomentosa*), a traditional medicinal plant widely distributed in southern China and Southeast Asia, has a documented history to treat hepatitis, gastralgia, diarrhea, and to boost the immune system [11,12,13,14]. The fruit of this plant (RTF) contains abundant phenolic and flavonoid compounds, whose antioxidant and anti-inflammatory activities are well-characterized [15,16,17,18,19]. In contrast, the polysaccharides from RTF (RTFP) have received less attention, though they may possess significant immunomodulatory potential. Previous reports describe RTF polysaccharides primarily as neutral heteroglycans or mannose-based polymers [20,21,22]. Nevertheless, the detailed structure and immunomodulatory activity of an acidic, arabinan–rich polysaccharide from this source remain uninvestigated, creating a notable gap in the structure–activity relationship profile of RTFP.

In this study, a novel acidic polysaccharide from RTF (RTFP-2b) was isolated and purified. The monosaccharide composition, glycosidic linkage patterns, and complex structures of the polysaccharides were analyzed by gas chromatography-mass spectrometry (GC–MS), high-performance anion-exchange chromatography with pulsed amperometric detection (HPAEC-PAD), Fourier transform infrared spectroscopy (FT-IR), and nuclear magnetic resonance (NMR) spectroscopy. The immunomodulatory properties of RTFP-2b were evaluated by monitoring its effects on cell activity, phagocytic capacity, nitric oxide (NO) production, and amounts of tumor necrosis factor-α (TNF-α), interleukin-6 (IL-6), and interleukin-1β (IL-1β) in lipopolysaccharide (LPS)-stimulated RAW264.7 cells. Finally, to explore its regulatory mechanisms, its effects on the nuclear factor kappa-B (NF-κB) pathway were determined. This research aims to fill the knowledge gap regarding RTF polysaccharides to leverage their potential as safe, multi-target immunomodulatory agents.

## 2. Results and Discussion

### 2.1. Separation and Purification of RTFP

**Figure 1:**
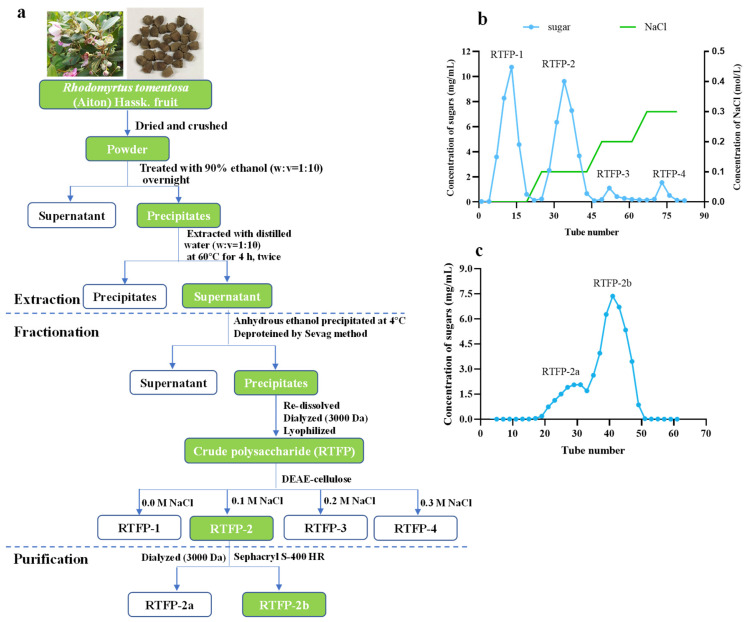
Extraction and purification of *R. tomentosa* fruit polysaccharide (RTFP). (**a**) Isolation and purification flow diagram (green boxes indicate the main procedural steps); (**b**) Elution curves of RTFP by chromatography on a DEAE-cellulose column showing four fractions (RTFP-1, RTFP-2, RTFP-3, and RTFP-4); (**c**) Elution curves of RTFP-2 by chromatography on a Sephacryl S-400 HR column showing a single symmetrical peak corresponding to the purified polysaccharide RTFP-2b.

After degreasing the RTF with anhydrous ethanol, the material was extracted with water at 60 °C, precipitated with alcohol, and then proteins were removed (Figure 1a). Then, the extract was concentrated and dried to acquire crude polysaccharides (RTFP), with a final yield of 4.2% (based on raw fruit dry weight). The RTFP was subsequently fractionated on a DEAE-cellulose column (26 mm × 400 mm) using distilled water at a flow rate of 4 mL/min, followed by elution with 0.1, 0.2, and 0.3 M NaCl. This process yielded four distinct fractions: RTFP-1, RTFP-2, RTFP-3, and RTFP-4 (Figure 1b). Of those, RTFP-2 had fewer peaks on the chromatogram and the highest total carbohydrate content. The polysaccharide fraction RTFP-2 was further purified via Sephacryl S-400 HR column (26 mm × 1000 mm) chromatography to remove low-molecular-weight impurities, yielding a homogeneous polysaccharide fraction. The main peak was collected, and represented a homogenous polysaccharide designated as RTFP-2b (Figure 1c), with a total carbohydrate content of 95.2% as determined by the phenol sulfuric acid method. The fractions corresponding to the main peak were collected for further analysis. The successful isolation of the RTFP-2b carbohydrate, which exhibited high purity and a distinct elution profile, provided a foundation for subsequent structural and bioactivity studies. Its high purity ensured that the observed effects in bioactivity assays originated from the polysaccharide itself, and not from co-extracted impurities.

### 2.2. Structure Characterization

#### 2.2.1. Purity and Relative Molecular Mass of RTFP-2b

**Figure 2:**
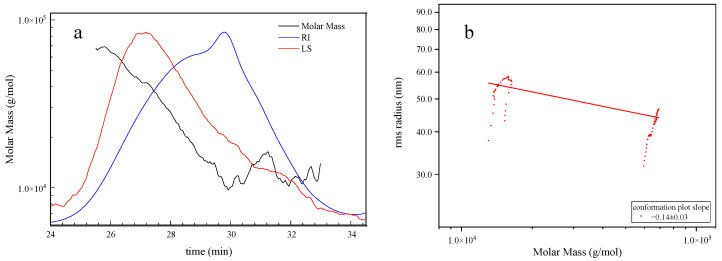
Absolute molecular weight analysis graph (**a**) and molecular conformation diagram (**b**). (**a**) Red line shows trend in multiangle laser light scattering signal of RTFP-2b with retention time; blue line shows trend in difference signal (RI) of RTFP-2b; black line represents molecular weight fitted by both signals. (**b**) Horizontal axis represents log (molar mass), vertical axis shows log (root mean square radius; RMS radius); and slope of this relationship is an indicator of molecular conformation.

The UV spectrum reveals characteristic absorption peaks for nucleic acids and proteins at 260 nm and 280 nm, respectively, enabling the assessment of impurities in polysaccharides through UV scanning [23,24]. As shown in Figure S1, RTFP-2b displayed no absorption peaks within the 200–400 nm range of the UV–vis spectrum in contrast to the blank control, indicative of the absence of significant nucleic acid and protein impurities. The bioactivity of polysaccharides is related to their molecular weight distribution, monosaccharide composition, and degree of branching. The molecular weight of RTFP-2b was determined using size exclusion chromatography-multi-angle laser light scatterer-differential refractive index detector (SEC-MALLS-RI). As shown in Figure 2a, the chromatogram displayed a relatively symmetrical peak with a minor shoulder, indicating of the homogeneity of RTFP-2b [25]. Based on the molecular weight parameters in Table S1, accounting for sample variability, RTFP-2b exhibited a weight-average molecular weight (Mw) of 22.995 kDa, a number-average molecular weight (Mn) of 16.794 kDa, and a polydispersity index (Mw/Mn) of 1.369. These results indicate that RTFP-2b exists as a moderately dispersed polymer [26]. The conformation plot (Figure 2b) had an average slope of −0.14 ± 0.03, reflecting the molecular shape and flexibility of RTFP-2b in solution. These results confirm that RTFP-2b is a relatively homogeneous polysaccharide with a well-defined molecular weight.

#### 2.2.2. Monosaccharide Composition

**Figure 3:**
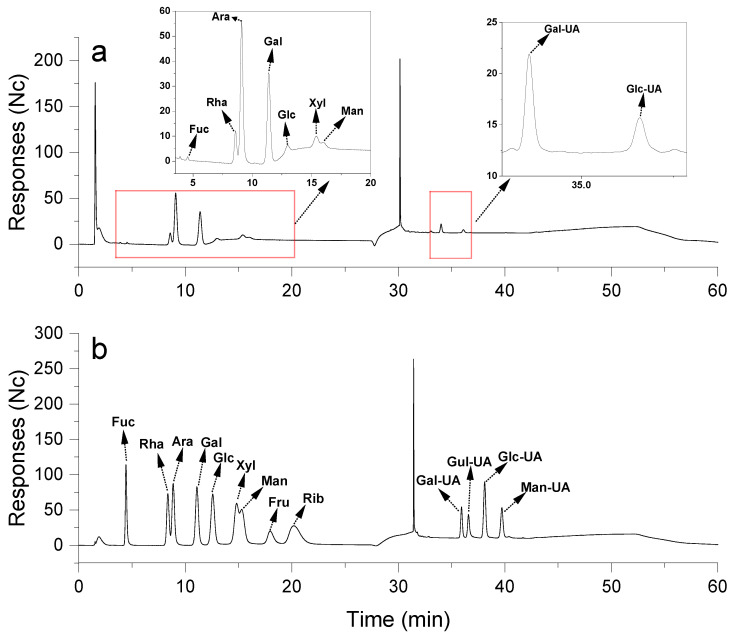
Monosaccharide composition analysis of RTFP-2b. (**a**) HPAEC-PAD chromatogram of RTFP-2b after complete acid hydrolysis; (**b**) chromatogram of monosaccharide standards.

Understanding the monosaccharide composition is crucial for deciphering the structural features, characteristics, and biological functions of polysaccharides, and for ensuring the quality of functional polysaccharides [27]. After the complete acid hydrolysis of RTFP-2b, its monosaccharide composition was analyzed by HPAEC-PAD, with monosaccharide standards for comparison. As shown in Figure 3 and Table 1, the analyses revealed the main constituents of RTFP-2b: namely Fucose (Fuc), rhamnose (Rha), arabinose (Ara), galactose (Gal), glucose (Glc), xylose (Xyl), mannose (Man), galacturonic acid (Gal-UA), glucuronic acid (Glc-UA), in a mole ratio of 0.59:9.37:38.68:21.86:3.5:5.28:2.66:14.83:3.22. The results suggested RTFP-2b contained a greater variety of sugar types. The main sugar constituents were Ara and Gal, and the significant presence of Gal-UA (14.83%) confirmed the acidic nature of the polysaccharide. Compared to polysaccharides from other fruits, RTFP-2b exhibits a distinct monosaccharide profile. For instance, an acidic pectin from *Gardenia jasminoides* fruit is predominantly composed of GalUA, with only minor quantities of neutral sugars like Ara and Gal [28]. The notably high Ara content (38.68%) in RTFP-2b is similar to that of some pectic arabinans or arabinogalactans, which are recognized for their immunomodulatory properties. This composition, characterized by the co-dominance of Ara and Gal along with substantial GalUA, contrasts with the homogalacturonan-rich pectins found in fruits like *Schisandra chinensis* and the polysaccharides from *Litchi chinensis* pericarp, which exhibit distinct neutral sugar profiles [29,30]. Ara-rich polysaccharides typically exhibit immunostimulatory effects, while Gal-UA, a component of pectin-like structures, often demonstrates anti-inflammatory activity through interacting with immune cell surfaces and modulating receptor signaling [7,31]. The presence of these constituents suggested that RTFP-2b may have good biological activity. Further research should establish whether RTFP-2b is localized in the pulp, peel, or seeds, and whether it serves particular functions in the fruit.

#### 2.2.3. FT-IR Spectroscopy Analysis

**Figure 4:**
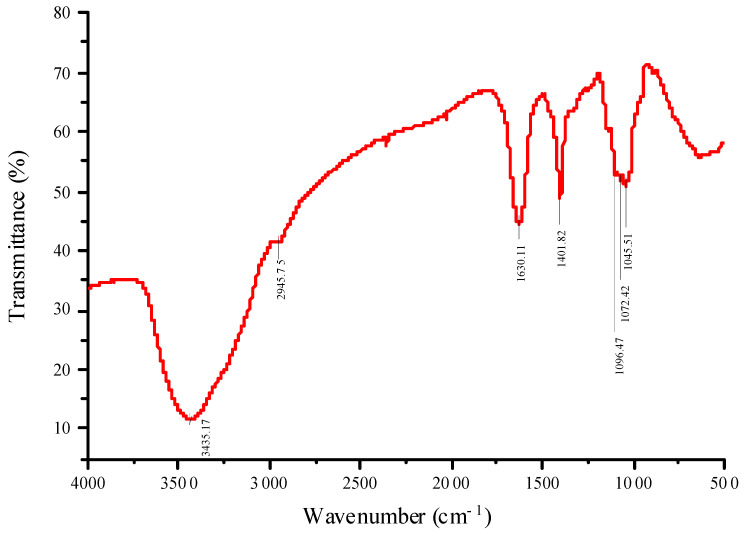
FT-IR spectrum of RTFP-2b. Characteristic absorption peaks at 3435.17 cm^−1^, 2945.75 cm^−1^, and 1630.11 cm^−1^ confirm the presence of hydroxyl groups, C–H stretching, and uronic acid carboxyl groups, respectively. Additional peaks between 1100 and 1000 cm^−1^ correspond to pyranose and furanose rings.

Next, FT-IR spectroscopy was conducted to determine the infrared absorption spectrum of RTFP-2b in the range of 4000–500 cm^−1^ (Figure 4). The strong absorption peak near 3435.17 cm^−1^ corresponded to the O-H stretching vibration, characteristic of carbohydrates [32]. The absorption peak at 2945.75 cm^−1^ was attributed to the stretching vibration of C-H [33]. The peak at 1630.11 cm^−1^ was assigned to the asymmetric and symmetric stretching vibration absorption peak of C=O in uronic acid carboxyl groups, indicating the presence of Gal-UA and Glc-UA [7]. Uronic acid confers a negative charge that increases polysaccharide solubility and promotes electrostatic interactions with immune cell receptors [25]. The absorption peak at 1401.82 cm^−1^ was associated with the bending vibrational mode of C-H bonds [34]. Additionally, the signal peaks at 1100–1000 cm^−1^ were associated with the stretching vibrations of C-O-H (ring vibration) and C-O-C (glycosidic bond), indicating the presence of both pyranose and furanose rings in RTFP-2b [35,36].

#### 2.2.4. XRD Analysis

X-ray diffraction analysis is a key technique for analyzing the structure of polymers and compounds, and primarily reveals the crystalline state of substances [37]. The physical properties of polysaccharides, such as flexibility and solubility, are closely linked to their crystalline structure. Typically, a broad rounded peak suggests a non-crystalline structure, whereas a sharp diffraction peak signifies a crystalline structure. As shown in Figure S2, the XRD spectrum within the 2θ range of 0 to 60° displayed no significant strong diffraction absorption peaks, but instead showed a diffuse scattering pattern with minor peaks. Notably, a prominent broad diffraction peak was present in the 15° to 25° diffraction angle range (Figure S2), indicative of a predominately amorphous structure of RTFP-2b, with a small proportion of crystalline regions. Its amorphous nature confers high water solubility. The limited crystalline regions likely enhance structural stability during storage [38]. Thus, the dual structure of RTFP-2b confers distinct pharmaceutical benefits [34].

#### 2.2.5. SEM Analysis of RTFP-2b

**Figure 5:**
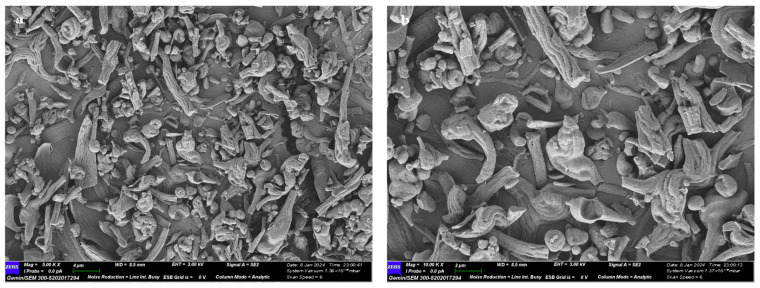
Scanning electron micrographs of RTFP-2b ((**a**) 5000× magnification; (**b**) 10,000× magnification). The images reveal an irregular, block-like morphology characterized by distinct striations and a microporous texture.

SEM was employed to characterize the micromorphological features of RTFP-2b. These analyses allowed for detailed visualization of its surface architecture at micrometer and sub-micrometer resolutions. As shown in Figure 5, the micrographs captured at 5000× and 10,000× magnifications revealed an irregular block-like morphology of RTFP-2b, with distinct striations. This morphology is consistent with a moderately dispersed polymer, as indicated by RTFP-2b’s polydispersity index of 1.369, and reflects its amorphous structural organization [39]. The lack of crystalline domains, consistent with the XRD results, confirmed the amorphous nature of RTFP-2b, which promotes water solubility and bioavailability. The microporous texture implied a substantial capacity for hydration and a large specific surface area, properties frequently associated with a high water-holding capacity [37]. The compact, aggregated appearance implies strong cohesive forces within the polysaccharide matrix, which likely arise from extensive hydrogen bonding and van der Waals interactions [26]. This structure denotes a stable polymeric network.

#### 2.2.6. Methylation Analysis of RTFP-2b

To further elucidate the sugar residues of RTFP-2b, methylation analysis was performed, and the detailed linkage patterns were analyzed by GC–MS. The supplementary data include the total ion chromatogram (Figure S3) and tandem mass spectra (Figure S4). Details of the methylation analysis of RTFP-2b are shown in Table 2. The analysis revealed 18 types of linkages across all sugar residues in RTFP-2b. Four linkage patterns were identified for Ara: Ara*f*-(1→ (20.89%), →5)-Ara*f*-(1→ (9.99%), →3)-Ara*f*-(1→ (6.47%), and →2,5)-Ara*f*-(1→ (1.24%). The total molar proportion of Ara-related linkages was approximately 38.59%, closely matching the Ara content (38.68%) determined by HPAEC-PAD. The combined proportion of Gal-related linkages (Gal*p*-(1→ [6.76%], →3)-Gal*p*-(1→ [2.77%], →4)-Gal*p*-(1→ [10.26%], →6)-Gal*p*-(1→ [1.65%], and →3,4,6)-Gal*p*-(1→ [2.52%]) was 23.96%, similar to the Gal*p* content (21.86%) determined in the monosaccharide analysis. The minor discrepancies were likely due to partial degradation of Gal residues during methylation. Furthermore, the abundance of the →4)-α-Gal*p*A-(1→ linkage (16.66%) was consistent with the results of the monosaccharide composition analysis (Gal-UA content 14.83%), indicating that RTFP-2b is an acidic polysaccharide [8,31]. The backbone of RTFP-2b contains structural motifs of →4)-α-D-Gal*p*A, which are also found in fruit pectins from sources such as *Gardenia jasminoides* and *Schisandra chinensis* [28,30]. In RTFP-2b, however, these linear segments are integrated into a more complex and highly branched heteropolysaccharide structure. RTFP-2b, with its high proportions of terminal Ara*f* (20.89%) and →5)-Ara*f* (9.99%), indicate the presence of extensive arabinan or arabinan-containing side chains. This structural feature contrasts with the neutral side-chain architectures reported for other fruit polysaccharides [25,30]. Signals of Fuc were not apparent, probably because it accounted for a very low proportion of residues in RTFP-2b (Table 1). The detection of 1,4,5-tri-O-acetyl-2,3,6-tri-O-methyl galactitol, which corresponds to 1,4-linked Gal-UA, indicates that the principal backbone of the polysaccharides is composed of 1,4-linked Gal-UA residues. Additionally, 1,5-di-O-acetyl-2,3,4,6-tetra-O-methyl galactitol indicated terminal Gal-UA residues, and 1,4-di-O-acetyl-2,3,5-tri-O-methyl arabinitol corresponded to terminal Ara residues. The presence of 1,5,6-tri-O-acetyl-2,3,4-tri-O-methyl galactitol and 1,3,5-tri-O-acetyl-2,4,6-tri-O-methyl galactitol indicated 6-linked Gal residues and 3-linked Gal residues, respectively. The presence of 1,3,4-tri-O-acetyl-2,5-di-O-methyl arabinitol suggested 3-linked Ara in side chains, and the presence of 1,5-di-O-acetyl-6-deoxy-2,3,4-tri-O-methyl rhamnitol implied that Rha serves as a branch point or internal residue within the polysaccharide chain. Taken together, these results indicate that RTFP-2b was primarily composed of Ara, Gal, and Gal-UA, with a linear backbone and limited branching. These linkage patterns were further confirmed by NMR spectroscopy.

#### 2.2.7. NMR Spectroscopy Analysis

**Figure 6:**
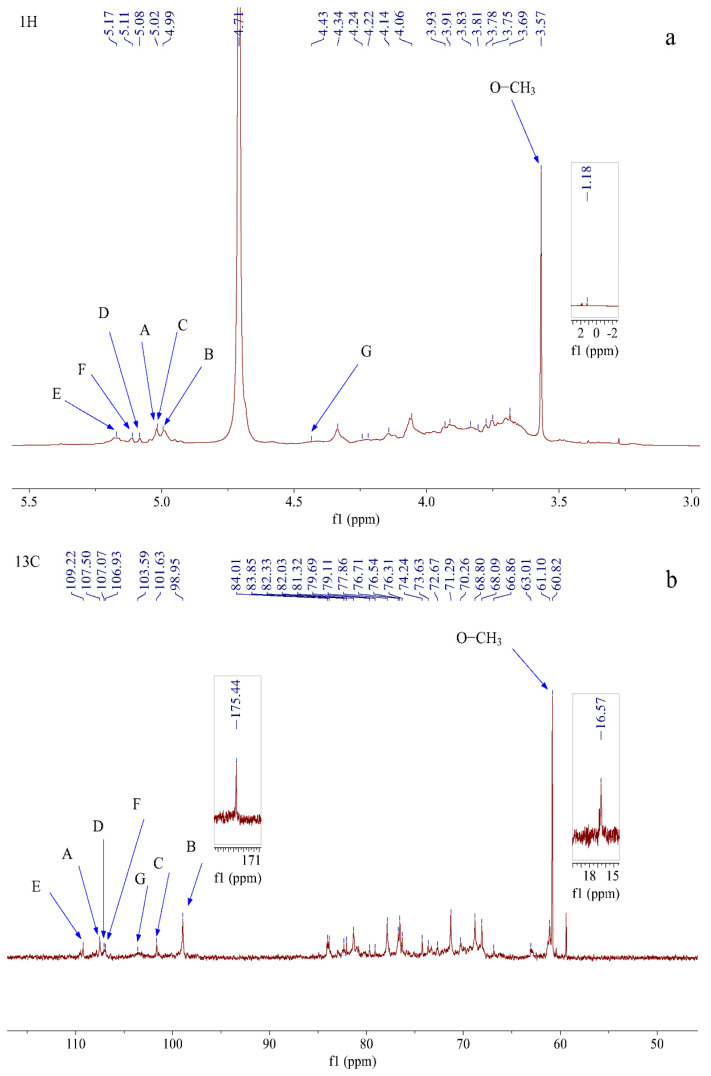

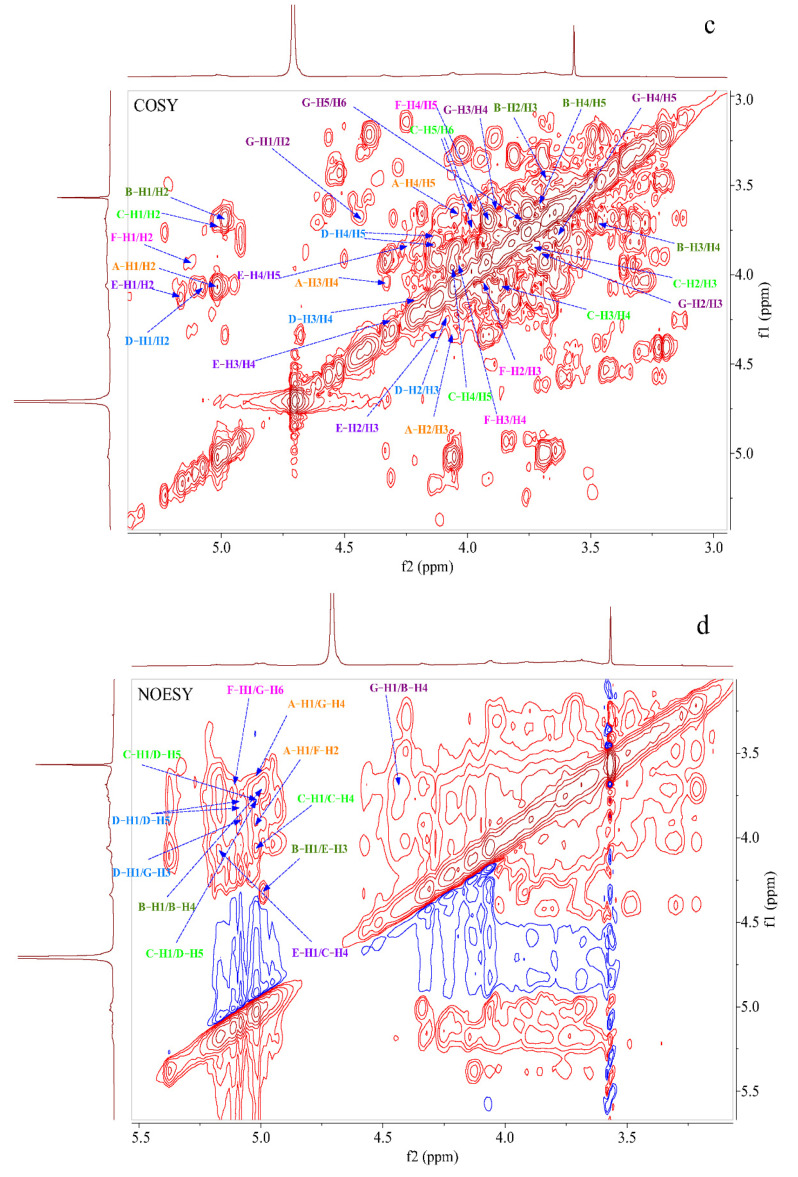

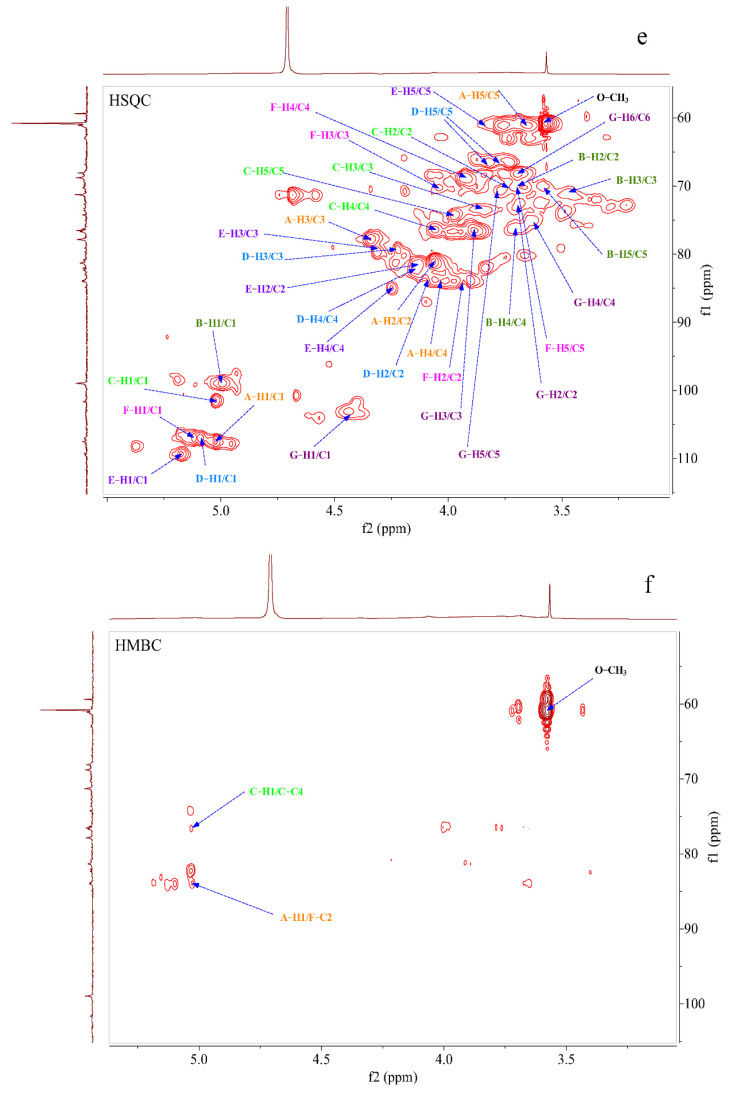
1D and 2D NMR spectra of RTFP-2b: ^1^H spectrum (**a**), ^13^C spectrum (**b**), COSY spectrum (**c**), NOESY spectrum (**d**), HSQC spectrum (**e**), HMBC spectrum (**f**). The meaning of capital letters in the figure: (A: α-L-Ara*f*-(1→; B: →4)-α-D-Gal*p*A-(1→; C: →4)-α-D-Gal*p*-(1→; D: →5)-α-L-Ara*f*-(1→; E: →3)-α-L-Ara*f*-(1→; F: →2)-α-L-Rha*p*-(1→; G: →3,4,6)-β-D-Gal*p*-(1→. “A-H1/H2” in part “c” means the H1/H2 cross-peak of residue A; “A-H1/F-H2” in part “d” means the cross-peak between residue A-H1 and F-H2; “A-H1/C1” in part “e” means the H1/C1 cross-peak of residue A; “A-H1/F-C2” in part “f” means the cross-peak between residue A-H1 and F-C2.

The structure of RTFP-2b was further analyzed by NMR. As shown in Figure 6, the complete NMR characterization including 1D (^1^H and ^13^C NMR) and 2D-NMR spectra (COSY, NOESY, HSQC, HMBC) provided critical structural information about RTFP-2b. Through ^1^H-NMR, the conformation of glycosidic bonds was clarified [40,41]. Typically, a signal in the 4.8 ppm region in the ^1^H-NMR spectrum is indicative of the α-configuration, and signals in the δ 4.3–4.8 ppm region are indicative of the β-configuration [8,42]. In the ^1^H NMR spectrum shown in Figure 6a, the proton signals of RTFP-2b were primarily concentrated in the range of δ 3.0–5.5 ppm. Within the anomeric proton region (δ 4.3–5.4 ppm), multiple coupled signal peaks were identified, indicating the presence of various sugar residues in RTFP-2b. The chemical shifts corresponding to the anomeric protons were δ 4.43, 4.99, 5.02, 5.02, 5.08, 5.11, and 5.17 ppm, and the non-anomeric proton signals were mainly distributed in the region of δ 3.1–4.2 ppm. Because of severe overlapping of some signals, further assignments of the chemical shifts for H2–H6 of each sugar residue were performed using COSY and HSQC spectra. The ^13^C NMR spectrum (Figure 6b) displayed characteristic anomeric carbon signals between δ 98.95 and 109.22 ppm [40]. Signals at δ 1.18 ppm in the ^1^H-NMR spectrum and δ 16.57 ppm in the ^13^C NMR spectrum were assigned to the CH_3_ group (C6) of Rha. The signal at δ 175.44 ppm in the ^13^C NMR spectrum confirmed the presence of Gal-UA and methylated Gal-UA, consistent with the monosaccharide composition results.

Combined with the cross-peaks observed in the HSQC spectrum (Figure 6e), the anomeric signals present in RTFP-2b were determined as follows: δ 5.02/107.5, 4.99/98.95, 5.02/101.63, 5.08/107.07, 5.17/109.22, 5.11/106.93, and 4.43/103.59 ppm, assigned to sugar residues A, B, C, D, E, F, and G, respectively. These findings establish the fundamental sugar composition and linkage patterns within RTFP-2b’s molecular architecture.

Taking sugar residue A as an example, the 1H-1H COSY spectrum of sugar residue A (Figure 6c) had correlation signals at δH 5.02/4.06 (H1-H2), 4.06/4.34 (H2-H3), 4.34/4.03 (H3-H4), and 4.03/3.65 (H4-H5), establishing the proton chemical shifts as δH 4.06 (H2), 4.34 (H3), 4.03 (H4), and 3.65 (H5). Additionally, the HSQC spectrum revealed cross-peaks at δ 5.02/107.5, 4.06/81.32, 4.34/77.8, 4.03/84.02, and 3.65/61.13, assigning the C2–C5 chemical shifts in residue A to 81.32, 77.8, 84.02, and 61.13 ppm, respectively. The downfield shift in C1 suggested O-1 substitution on the sugar ring [25]. Therefore, residue A was considered to be an Ara residue with the linkage type α-L-Ara*f*-(1→, which was mutually corroborated by the results of the monosaccharide composition and methylation analyses and chemical shifts reported in the literature [7,43]. By combining these results with data in the literature, the remaining signals for the other six sugar residues were attributed as follows: →4)-α-D-Gal*p*A-(1→ (B), →4)-α-D-Gal*p*-(1→ (C), →5)-α-L-Ara*f*-(1→ (D), →3)-α-L-Ara*f*-(1→ (E), →2)-α-L-Rha*p*-(1→ (F), →3,4,6)-β-D-Gal*p*-(1→ (G) [44,45,46]. Although the aldehydic acid of →4)-α-D-Gal*p*A-(1→ exists in the form of a methoxy ester group (-COOCH_3_), the signal peak surrounding δ 3.58/60.82 ppm originates from the carbon signal of O-CH_3_ [33]. Table 3 displays the chemical shift values for the seven glycosyl residues.

The chemical shifts in ^13^C and ^1^H for each sugar residue in RTFP-2b, along with HMBC and NOESY spectral data, revealed specific structural features and linkage patterns in RTFP-2b. The severe spectral overlap inherent to polysaccharide NMR analysis stems from limited chemical shift dispersion among similar sugar residues and from conformational flexibility that broadens the peaks. Furthermore, long-range HMBC correlations are frequently weak or absent owing to rapid relaxation and large coupling constants, complicating definitive linkage assignments. In this work, the HMBC spectrum provided limited long-range information; so, some glycosidic linkages were deduced mainly from NOESY correlations, which indicate spatial proximity rather than direct covalent bonds. The resulting structural model must therefore be regarded as tentative and requires further validation with complementary techniques. The HMBC spectrum (Figure 6f) had a cross-peak at δ 5.02/83.86 ppm, correlating residue A-H1 with F-C2. Correspondingly, the NOESY spectrum (Figure 6d) had a cross-peak at δ 5.02/3.93 ppm between residue A-H1 and F-H2, confirming the presence of an α-L-Ara*f*-(1→2)-α-L-Rha*p*-(1→ linkage fragment. Furthermore, the HMBC spectrum had a cross-peak at δ 5.02/76.38 ppm between residue C-H1 and C-C4. Within the NOESY spectrum, cross-peaks were observed at δ 5.02/4.06 ppm between residue C-H1 and C-H4, and at δ 5.02/3.78 ppm and δ 5.02/3.83 ppm between residue C-H1 to D-H5, indicating a →4)-α-D-Gal*p*-(1→4)-α-D-Gal*p*-(1→5)-α-L-Araf-(1→ linkage fragment. Because the HMBC spectrum was relatively weak and provided limited long-range correlation information, the other structural linkages were determined mainly based on the NOESY spectrum [47]. In the NOESY spectrum, cross-peaks were also observed at δ 4.99/3.7 ppm between residue B-H1 and B-H4, corresponding to a →4)-α-D-Gal*p*A-(1→4)-α-D-Gal*p*A-(1→ linkage fragment. Additional cross-peaks were present at δ 4.99/4.31 ppm between residue B-H1 and E-H3, indicating a →4)-α-D-Gal*p*A-(1→3)-α-L-Ara*f*-(1→ linkage fragment. Signals at δ 5.08/3.78 ppm and δ 5.08/3.83 ppm between residue D-H1 and D-H5, confirmed a →5)-α-L-Ara*f*-(1→5)-α-L-Ara*f*-(1→ linkage fragment. A cross-peak at δ 5.08/3.88 ppm between residue D-H1 and G-H3 suggested a →5)-α-L-Ara*f*-(1→3,4,6)-β-D-Gal*p*-(1→ linkage. The correlation at δ 5.02/3.63 ppm between H1 of sugar residue A and H4 of sugar residue G was assigned to a α-L-Ara*f*-(1→3,4,6)-β-D-Gal*p*-(1→ linkage. A signal at δ 5.17/4.06 ppm between H1 of sugar residue E and H4 of sugar residue C indicated a →3)-α-L-Ara*f*-(1→4)-α-D-Gal*p*-(1→ linkage. The cross-peak at δ 5.11/3.69 ppm between H1 of sugar residue F and H6 of sugar residue G indicated a →2)-α-L-Rha*p*-(1→3,4,6)-β-D-Gal*p*-(1→ linkage. Finally, a correlation at δ 4.43/3.7 ppm between H1 of sugar residue G and H4 of sugar residue B indicated a →3,4,6)-β-D-Gal*p*-(1→4)-α-D-Gal*p*A-(1→ linkage.

**Figure 7:**
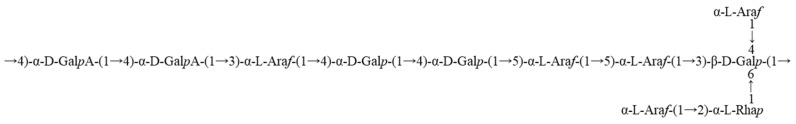
Proposed structure of RTFP-2b.

On the basis of the 1D and 2D NMR data, as well as the results of the methylation analysis, we propose a tentative structural model for RTFP-2b. In this model, the polysaccharide may consist of →4)-α-D-Gal*p*A-(1→, →4)-α-D-Gal*p*-(1→, and →5)-α-L-Ara*f*-(1→ potentially interconnected to constitute the backbone. The presence of the →5)-α-L-Ara*f* linkage within this proposed backbone is inferred primarily from NOESY correlations (e.g., δ 5.08/3.78 and δ 5.08/3.83 ppm, suggesting a →5)-α-L-Ara*f*-(1→5)-α-L-Ara*f*-(1→ sequence); however, its exact sequential position and confirmation as an integral part of a linear backbone, as opposed to a long side chain, would benefit from additional evidence. The branches are proposed to be mainly connected by α-L-Ara*f*-(1→ at the O-4 position of the sugar residue →3,4,6)-β-D-Gal*p*-(1→, and α-L-Ara*f*-(1→ and →2)-α-L-Rha*p*-(1→ were interconnected and connected at the O-6 site of →3,4,6)-β-D-Gal*p*-(1→. Figure 7 shows the proposed structure of RTFP-2b, represents a plausible model integrating the identified linkage motifs and NOESY correlations. It should be noted that while the methylation and monosaccharide composition data are consistent with the presence of these residues and linkages, the precise sequence and some connectivity details, particularly those relying on weaker NOESY correlations, require further confirmation. This structure differs from previously reported RTF polysaccharides characterized by Man*p*-based backbones [20]. The results of this study reveal previously unknown structural features and motifs of RTF polysaccharides, advancing our understanding of their molecular composition. The structural data allow for systematic comparison of structure–activity relationships through analysis of shared and divergent structural motifs.

### 2.3. Immunomodulatory Activity

#### 2.3.1. Effects of RTFP-2b on the Viability of LPS-Induced RAW264.7 Cells

**Figure 8:**
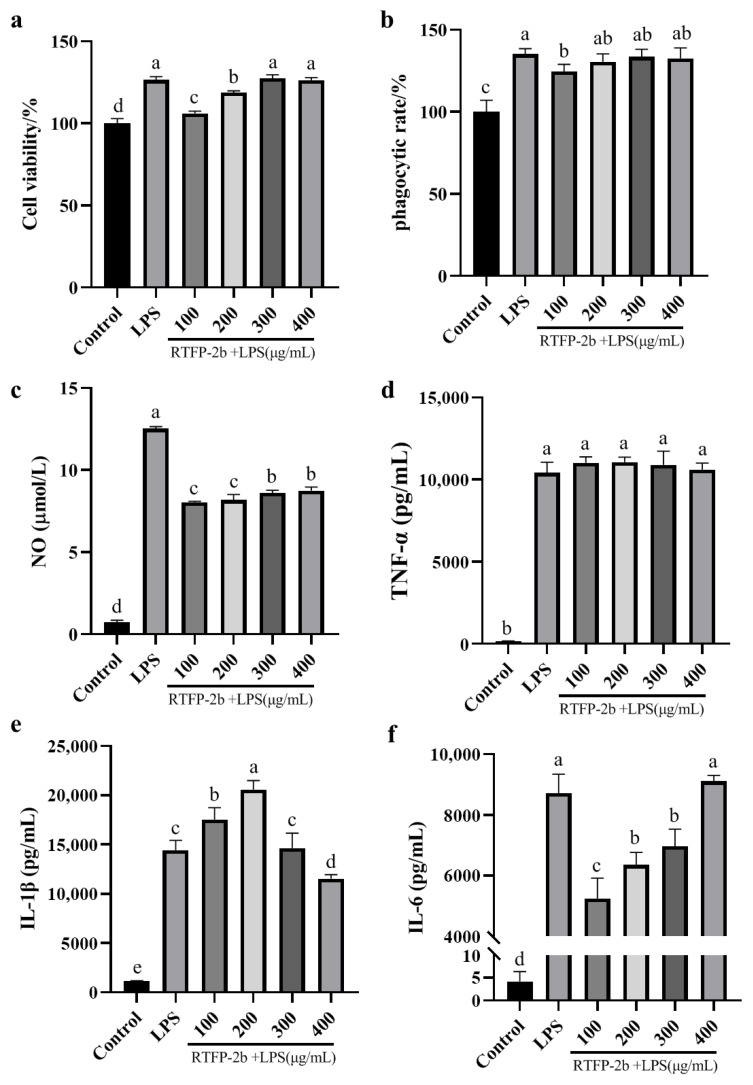
Effects of RTFP-2b on cell activity (**a**), phagocytic rate (**b**), NO yield (**c**), and levels of TNF-α (**d**), IL-1β (**e**), IL-6 (**f**) in LPS-stimulated RAW264.7 cells. Cells were exposed to various concentrations of RTFP-2b (100, 200, 300, 400 μg/mL) with or without the presence of 1 μg/mL LPS for 24 h. Data are mean ± SD (*n* = 3). Different letters (a–e) above bars indicate significant differences among treatment groups (*p* < 0.05, one-way ANOVA). All LPS-induced groups (including RTFP-2b treated) differed significantly from the control group (*p* < 0.05).

Lipopolysaccharide, a key constituent of Gram-negative bacterial outer membranes, potently activates macrophages and is significant for immunomodulation, vaccine development, and studies of disease mechanisms [48,49]. Our research assessed the immunomodulatory effects of RTFP-2b administered to LPS-stimulated RAW264.7 cells. The CCK-8 method was used to evaluate the effect of RTFP-2b on the viability of LPS-stimulated RAW264.7 cells. As shown in Figure 8a, compared with the blank control group, the LPS-stimulated RAW264.7 cells showed markedly increased metabolic activity. The addition of RTFP-2b at concentrations of 100 and 200 μg/mL significantly inhibited the metabolic activity of LPS-induced cells, although their activity level was still higher than that of the blank group. Conversely, application of RTFP-2b at concentrations of 300 and 400 μg/mL did not significantly affect the metabolic activity of LPS-stimulated RAW264.7 cells. Consequently, RTFP-2b solutions with concentrations of 100, 200, 300, and 400 μg/mL were selected for subsequent experiments, a range consistent with typical doses used for in vitro evaluations of polysaccharide bioactivity in the literature [7,50,51].

#### 2.3.2. Effects of RTFP-2b on the Phagocytic Capacity of LPS-Induced RAW264.7 Cells

The phagocytic activity of macrophages is the first line of nonspecific immune defense against invading pathogens. Once the pathogen is engulfed, macrophages induce specific immunoreactions that activate the inflammatory response. Thus, their phagocytic activity is a key indicator of the level of systematic immune protection [52]. Research has shown that polysaccharides derived from natural plants can promote phagocytic activity, thereby strengthening immune regulation [53]. Figure 8b shows the effect of RTFP-2b polysaccharides on the phagocytic activity of LPS-induced RAW264.7 cells. Utilizing the blank group as a reference with a 100% phagocytosis rate, RAW264.7 cells showed a notable increase in phagocytic activity following LPS induction. Compared with the LPS-stimulated group, the application of RTFP-2b at concentrations of 200, 300, and 400 μg/mL had minimal effects on the phagocytic activity of these cells, but application of RTFP-2b at 100 μg/mL decreased the magnitude of the LPS-induced increase, although the phagocytosis rate still reached 124.5% in this treatment. The phagocytic activity was significantly higher in the RTFP-2b-treated cells than in the control. This enhancement aligns with the known function of certain polysaccharide structures, particularly those rich in Ara and Gal side chains, as mild immune stimulants [53].

#### 2.3.3. Effects of RTFP-2b on the Levels of NO, TNF-α, IL-1β, and IL-6 in LPS-Stimulated RAW264.7 Cells

**Figure 9:**
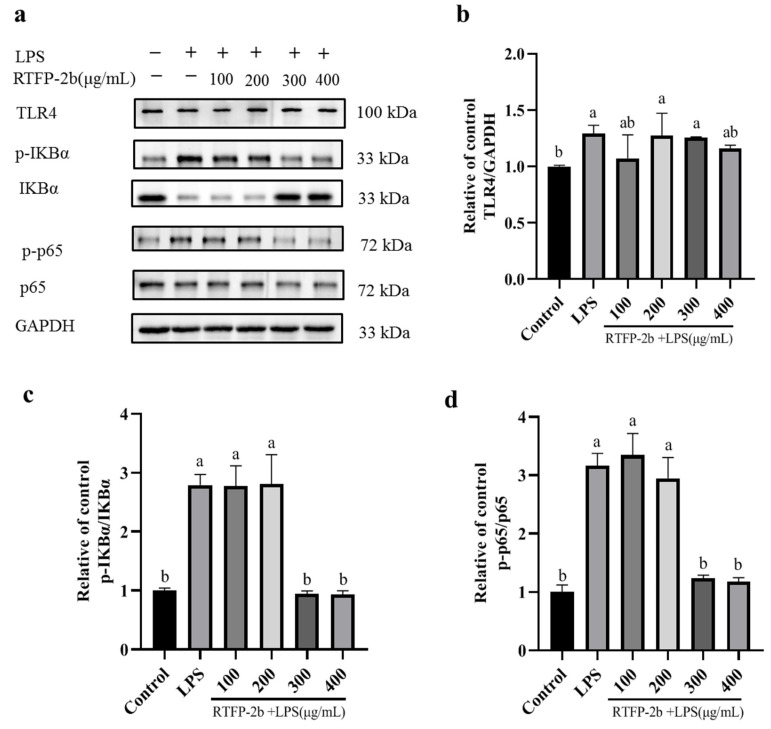
Western blot image (**a**) showing the effects of administration of RTFP-2b on the levels of TLR4 (**b**), p-IκBα/IκBα (**c**), and p-p65/p65 (**d**) in LPS-stimulated RAW264.7 cells. Cells were exposed to various concentrations of RTFP-2b (100, 200, 300, 400 μg/mL) with or without the presence of 1 μg/mL LPS for 24 h. Data are mean ± SD (*n* = 3). Different letters (a–b) above bars indicate significant differences among treatment groups (*p* < 0.05, one-way ANOVA). All LPS-induced groups differed significantly from the control group (*p* < 0.05).

Nitric oxide functions as a cellular signal transduction mediator. It stimulates macrophages to trigger nonspecific immunity, boosts their metabolism, and reinforces their phagocytic activity [50]. Thus, the NO level is a critical indicator of the strength of inflammatory reactions. Cytokines are also important indicators; they are secreted by macrophages during immune reactions and engage in the immune response and modulate macrophage functions [7,8]. Among these, TNF-α is the most significant inflammatory mediator. IL-1β is a key pro-inflammatory cytokine involved in disease resistance, and IL-6 is an important component of host defense, with several roles in immunological responses. Studies have shown that polysaccharides isolated from plants can inhibit the increases in TNF-α, IL-1β, IL-6, INF-γ, and NO in LPS-stimulated RAW264.7 cells [8,54]. Therefore, the effects of RTFP-2b on the levels of immunological indicators in LPS-stimulated RAW264.7 cells were investigated. As shown in Figure 8c–f, the levels of LPS, NO, TNF-α, IL-1β, and IL-6 were significantly higher in LPS-stimulated RAW264.7 cells than in the controls, indicating that LPS activated inflammation. Compared with the LPS-stimulated group, the cells treated with RTFP-2b showed significantly reduced NO production (*p* < 0.05), although the levels were still higher than those in the untreated control (Figure 8c). The level of TNF-α was slightly higher in the RTFP-2b-treated groups than in the LPS-stimulated group (*p* > 0.05) (Figure 8d). The highest IL-1β level was in the 200 μg/mL RTFP-2b treatment group, and declined at higher doses (Figure 8e), whereas the IL-6 levels increased with increasing concentrations of RTFP-2b (Figure 8f). This heterogeneous cytokine response implies context-specific immunomodulatory effects, rather than generalized suppression, consistent with the selective inhibition of the NF-κB pathway detected in cells treated with 300–400 μg/mL RTFP-2b (Figure 9). The concentration-dependent modulation of cytokine profiles indicated that RTFP-2b acts as a dual-phase immunomodulator, with lower doses (≤200 μg/mL) enhancing phagocytosis and IL-1β production through partial agonist activity, and higher doses (≥300 μg/mL) inhibiting NF-κB-mediated inflammation. This behavior corresponds to its structural transition; that is, the flexible side chains (Ara*f*/Rha*p*) interact with surface receptors at low concentrations, whereas the Gal*p*A-rich backbone domains competitively block intracellular signaling pathways at elevated concentrations [7,25]. This dose-dependent immunomodulatory behavior, particularly the suppression of pro-inflammatory mediators at higher concentrations mediated through NF-κB inhibition, occurs in other acidic fruit polysaccharides [28,30].

#### 2.3.4. Effects of RTFP-2b on the Levels of TLR4, p-p65/p65, and p-IκBα/IκBα in LPS-Induced RAW 264.7 Cells

The NF-κB signaling pathway plays a crucial role in inflammatory and immune responses by mediating the release of NO and cytokines in macrophage cells [8,50]. The key constituents of this pathway are IκBα, p65, and TLR4. Signaling in the NF-κB pathway is initiated by TLR4, which recognizes pathogen-associated molecular patterns and triggers downstream signaling events. IκBα acts as an inhibitor of NF-κB, while p65 is a primary transcriptional activator. Once TLR4 is stimulated, the IκB kinase (IKK) complex is activated, leading to the phosphorylation of IκBα. This phosphorylation event results in the ubiquitination and degradation of IκBα, enabling NF-κB (specifically, the p65/p50 dimer) to translocate into the nucleus and promote the expression of genes related to inflammation. By assessing the expression level and phosphorylation status of these key molecules, valuable insights into the activation state of the NF-κB signaling pathway and its involvement in inflammatory and immune responses can be obtained. In other studies, purified acid polysaccharides isolated from *Pyrus sinkiangensis* ‘Yu’ were found to downregulate IKK, IκBα, NF-κB, and p65 and upregulate TLR2 and TLR4 in lung tissues [51]. Similarly, a polysaccharide isolated from angelica was found to reduce the yield of inflammatory mediators (NO, TNF-α, IL-6, and IL-1β) while downregulating TLR4 and genes encoding several pro-inflammatory chemokines. In addition, the angelica polysaccharide inhibited the NF-κB signaling pathway by decreasing the phosphorylation of IκBα, p65 [55]. Higher LPS levels activate the NF-κB pathway, triggering the release of cytokines that impair intestinal barrier integrity. As a result, LPS can enter the circulatory system and trigger systemic chronic inflammatory reactions to further aggravate immune disease.

**Figure 10:**
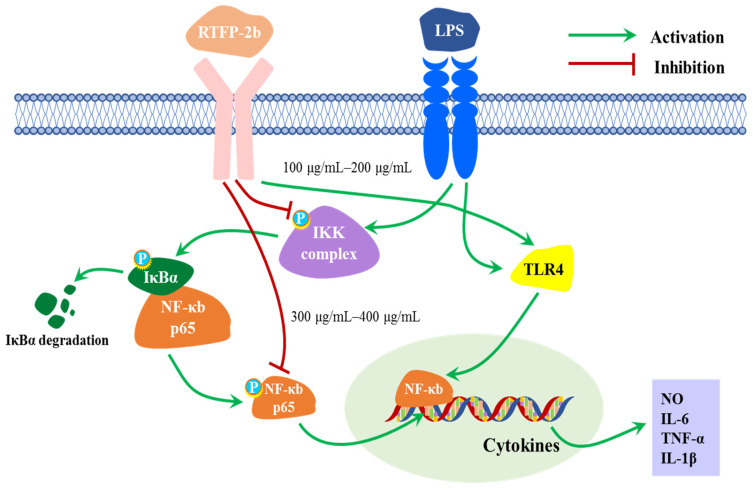
Potential mechanism of the immunomodulatory effect of RTFP-2b in LPS-stimulated RAW264.7 cells. At higher concentrations (300–400 μg/mL), RTFP-2b inhibits the phosphorylation of IκBα and p65, which blocks NF-κB nuclear translocation and reduces the expression of pro-inflammatory mediators. At lower concentrations (100–200 μg/mL), it exhibits partial immunostimulatory effects, such as enhanced phagocytosis and IL-1β production.

In this study, the levels of TLR4 and NF-κB and the ratios of p-p65/p65 and p-IκBα/IκBα in LPS-induced cells were determined (Figure 9a–d). Compared with the blank group, the LPS-stimulated group showed significantly increased levels of TLR4 and higher p-p65/p65 and p-IκBα/IκBα ratios (*p* < 0.05). Notably, LPS stimulated the NF-κB signaling pathway via the phosphorylation of TLR4, p65, and IκBα, leading to inflammation. Upon treatment with RTFP-2b at 100–200 μg/mL, the level of TLR4 was lower than that in the LPS-induced group. Treatment with RTFP-2b concentrations of 100 and 200 μg/mL did not significantly alter the p-p65/p65 and p-IκBα/IκBα ratios, relative to those in the positive control group. Interestingly, in cells treated with RTFP-2b at 300 and 400 μg/mL, the p-p65/p65 and p-IκBα/IκBα ratios markedly decreased to almost the same as those in the blank. The reduced p-IκBα/IκBα ratio indicated that IKK activity was inhibited, leading to decreased phosphorylation of IκBα. The reduction in p-p65/p65 and p-IκBα/IκBα ratios in cells treated with RTFP-2b at 300–400 μg/mL indicated that the NF-κB pathway was inhibited by RTFP-2b at higher concentrations. However, this molecular effect did not consistently translate to cytokine modulation, suggesting that there were temporal delays in downstream signaling or concentration-dependent regulatory mechanisms. These findings suggest a potential mechanism of the immunomodulatory effect of RTFP-2b at higher doses. The modest effect on TLR4 expression, in contrast to the strong inhibition of downstream phosphorylation events (IκBα and p65), suggests that RTFP-2b acts intracellularly by interfering with IKK complex activation, rather than solely by blocking the binding of LPS to TLR4 extracellularly. Further research is needed to confirm this explanation for the immunomodulatory effect. On the basis of the results of this study, the potential mechanism of RTFP-2b’s immunomodulatory effect on LPS-induced RAW264.7 cells is proposed (Figure 10).

Specific structural regions significantly influence macrophage activation. As reported in other studies, the Gal content in polysaccharides is positively correlated with the anti-inflammatory activity of macrophages [56], and polysaccharides rich in Gal-UA demonstrate notable anti-inflammatory and immunomodulatory effects [7,57]. Polysaccharides from fruits containing a →4)-α-D-GalpA-(1→ backbone can remarkably reduce inflammatory cytokine levels [25,58]. Moreover, polysaccharides with molecular weights between 10 and 1000 kDa exhibit enhanced immunomodulatory capacity [59]. Accordingly, the immunomodulatory effect of polysaccharides is affected by diverse structural characteristics, such as glycosidic linkages, monosaccharide structure, and branching patterns. The immunomodulatory properties of RTFP-2b can be attributed to its molecular weight (22.995 kDa) and the presence of specific structural features such as →4)-α-D-GalpA-(1→. However, the innovation of RTFP-2b extends beyond these common active motifs to its unique composite structure, which integrates a GalUA-rich backbone with substantial neutral sugar side chains, particularly arabinans. This structural complexity and the resulting functional nuance distinguish RTFP-2b as a novel acidic heteropolysaccharide from *R. tomentosa* fruit, exhibiting characteristics that differ from polysaccharides found in well-studied fruits such as amla [48], and litchi [29].

The immunomodulatory activity of polysaccharides is intricately linked to their structural features. The dual-phase, dose-dependent behavior of RTFP-2b stems from its unique composite architecture. At lower concentrations, the flexible Araf and Rhap side chains may act as mild agonists for pattern recognition receptors such as TLR4, thereby potentiating phagocytosis and IL-1β production. At higher concentrations, however, the density packed galacturonan backbone dominates the interaction. We propose a mechanistic model in which high-dose RTFP-2b exerts its immunomodulatory effect primarily by intracellularly disrupting the NF-κB signaling cascade, potentially at the level of the IKK complex. This interpretation is supported by the marked inhibition of IκBα and p65 phosphorylation without a corresponding drastic reduction in TLR4 levels (Figure 9). The anionic nature of the Gal-UA residues may be essential for this intracellular activity, possibly by interfering with protein–protein interactions within the signalosome. This mechanism distinguishes RTFP-2b from polysaccharides that function solely as direct TLR4 antagonists, aligning with the complex, structure-dependent bioactivity reported for other acidic heteropolysaccharides [53,59]. The unexpected increase in IL-6 at higher concentrations, despite NF-κB inhibition, further suggests the involvement of alternative pathways such as MAPK or JAK-STAT, which may be preferentially activated by specific structural motifs in RTFP-2b and warrant future investigation [50,55].

### 2.4. Limitations and Future Perspectives

This study provides a comprehensive structural and bioactivity analysis, yet several limitations must be acknowledged. First, the proposed structural model for RTFP-2b, especially the placement of the →5)-α-L-Ara*f*-(1→ residue within the backbone, depends primarily on NOESY correlations with only limited support from HMBC data. The inherent complexity of polysaccharide NMR analysis, including signal overlap and weak long-range correlations, prevents definitive confirmation of all glycosidic linkages. Second, this research employed in vitro assays with LPS-stimulated RAW264.7 cells, while informative, these models cannot fully replicate the complex in vivo environment. Finally, the finding that RTFP-2b increased IL-6 secretion while inhibiting NF-κB phosphorylation at higher concentrations implies the involvement of signaling pathways beyond NF-κB, a possibility that requires further study.

To address these limitations and deepen the understanding of RTFP-2b, future work should pursue several objectives: validating the in vitro results using relevant in vivo immunomodulatory models to assess efficacy and safety; employing receptor blockade or siRNA knockdown to identify the primary receptors mediating its activity; investigating crosstalk with other pathways like MAPK and JAK/STAT to explain the complex cytokine response; and evaluating the prebiotic potential of RTFP-2b, considering that dietary polysaccharides can indirectly modulate immunomodulatory via the gut microbiota. Clarifying these mechanisms is crucial for harnessing the full therapeutic potential of RTFP-2b as a multifunctional immunomodulatory agent.

## 3. Materials and Methods

### 3.1. Materials and Reagents

Fully ripe RTF (approximately 1.0–1.5 cm in diameter) were collected from trees in Wuming, Guangxi Zhuang Autonomous Region, China (23°15′15″ N, 108°5′31″ E) in August 2023. All specimens exhibited uniform maturity, characterized by deep purple coloration, and had no physical damage or microbial contamination. The species was identified by Dr. Jianhua Sun (Guangxi Veterinary Research Institute, China). The fruit were air-dried and ground with a multi-function grinder (LX-02, Lixiang, Shanghai, China) at 25,000 rpm to achieve a powder with particle sizes in the range of 250–300 μm. The ground samples were dried to constant weight and stored in a desiccator until further use.

The DEAE-cellulose column (26 mm × 400 mm) was obtained from Sunresin New Materials Co., Ltd. (Xi’an, China) and Sephacryl S-400HR was obtained from the General Electric Company (Fairfield, CT, USA). Trifluoroacetic acid (TFA), dichloromethane (DCM), methanol, and ethanol were purchased from ANPEL Laboratory Technologies Inc. (Shanghai, China). The following standard monosaccharides were purchased from Sigma (St Louis, MO, USA): Fucose (Fuc), rhamnose (Rha), arabinose (Ara), galactose (Gal), glucose (Glc), xylose (Xyl), mannose (Man), galacturonic acid (Gal-UA), glucuronic acid (Glc-UA), fructose (Fru), ribose (Rib), guluronic acid (Gul-UA), and mannuronic acid (Man-UA). LPS, dimethyl sulfoxide (DMSO), sodium borodeuteride (NaBD_4_), sodium borohydride (NaBH_4_), 1-cyclohexyl-3-(2-morpholinoethyl) carbodiimide metho-*p*-toluenesulfonate (CMEC), imidazole and acetic anhydride were also obtained from Sigma.

Dulbecco’s modified Eagle’s medium (DMEM), penicillin, streptomycin, fetal bovine serum (FBS), and trypsin were purchased from Gibco BRL (Gaithersburg, MD, USA). The cell counting kit-8 (CCK-8) was obtained from Vazyme Biotech Co., Ltd. (Nanjing, China). The TNF-α, IL-6, and IL-1β enzyme-linked immunosorbent assay (ELISA) kits were obtained from Shanghai Jining Industrial Co., Ltd. (Shanghai, China), and the NO detection kit was obtained from Beyotime Biotech Inc. (Shanghai, China). Antibodies to Toll-like receptor 4 (TLR4), NF-κB p65, phosphorylated (p) NF-κB-p65, inhibitor of nuclear factor-κB alpha (IκB-α), and phosphorylated (p) IκB-α were obtained from Cohesion Biosciences (London, UK). All other reagents were of analytical grade.

### 3.2. Extraction and Purification

The ground RTF samples were crushed and treated with 90% ethanol overnight for degreasing and decolorization. The dried residues were subsequently extracted twice with hot water at 60 °C for 4 h using a material-to-water ratio of 1:10. The combined supernatants were concentrated and precipitated with four volumes of anhydrous ethanol at 4 °C to yield crude polysaccharides. This precipitate was then redissolved in water and deproteinized by the Sevag method [60], after which it was defatted with petroleum ether (Sinopharm Chemical Reagent Co., Ltd., Shanghai, China) and decolorized with AB-8 macroporous resin (Sunresin New Materials Co., Ltd., Xi’an, China). The solution was dialyzed (3000 Da cut-off membrane) against water, concentrated, and lyophilized to acquire the crude polysaccharide sample. The crude polysaccharide sample was loaded onto a DEAE-cellulose column (26 mm × 400 mm) and eluted with distilled water at 4 mL/min, followed by NaCl in a stepwise gradient (0, 0.1, 0.2, and 0.3 M NaCl). Carbohydrate content in the eluted fractions was monitored using the phenol-sulfuric acid method [25,61]. Four fractions (RTFP-1, RTFP-2, RTFP-3, and RTFP-4) were obtained. The main polysaccharide fraction RTFP-2 with the highest yield (25.2%) were collected, concentrated, dialyzed (3000 Da) against distilled water for 48–72 h. The polysaccharide solution was further purified through a Sephacryl S-400 HR column (26 mm × 1000 mm), using distilled water as the mobile phase at 1.0 mL/min. Total carbohydrates in the eluant were quantified using the phenol sulfuric acid method. This step yielded the pure polysaccharide, designated as RTFP-2b. The absence of endotoxin was confirmed using a Limulus amebocyte lysate (LAL) assay (Xiamen Bioendo Technology Co., Ltd., Xiamen, China).

### 3.3. Structural Characterization

#### 3.3.1. Chemical Composition Analysis

The total carbohydrate content was measured by the phenol sulfuric acid method with glucose as the standard [61]. Monosaccharide composition analysis of RTFP-2b was performed using an ICS-5000 high pressure ion chromatography system (Thermo Fisher Scientific, Waltham, MA, USA) equipped with an electrochemical detector [62]. Samples (approximately 5 mg) were hydrolyzed with 2 M TFA at 121 °C for 2 h in a sealed tube. After hydrolysis, the samples were dried under a flow of nitrogen gas, washed three times with methanol, and dried again. The residue was re-dissolved in distilled water and filtered through a 0.22 μm microporous membrane before measurement. The extracts were then analyzed by HPAEC on a CarboPac PA-20 anion-exchange column (3 × 150 mm; Dionex, Sunnyvale, CA, USA) with a pulsed amperometric detector (PAD; Dionex ICS 5000+ system). The mobile phase consisted of three solvent systems: A (distilled water), B (0.1 M NaOH), and C (0.1 M NaOH with 0.2 M NaAc). The gradient program ran at 0.5 mL/min with 5 μL injections: 95:5:0 (A: B: C) from 0 to 26 min, 85:5:10 from 26 to 42 min, 60:0:40 at 42.1 min, 60:40:0 from 52 min, returning to 95:5:0 from 52.1 to 60 min.

#### 3.3.2. Molecular Weight Determination

The homogeneity and molecular weight of various fractions of RTFP-2b were analyzed with a SEC-MALLS-RI detector [63]. Measurements of weight-average (Mw) and number-average (Mn) molecular weights, as well as polydispersity index (Mw/Mn), were performed in 0.1 M NaNO_3_ aqueous solution with 0.02% NaN_3_ containing 0.5% LiBr using a DAWN HELEOS-II system (Wyatt Technology, Santa Barbara, CA, USA) with two tandem columns at 60 °C. The mobile phase was delivered at a flow rate of 0.6 mL/min, and the system included a differential refractive index detector for simultaneous concentration measurements and dn/dc determination. The dn/dc value of samples in 0.1 M NaNO_3_ aqueous solution containing 0.02% NaN_3_ was measured as 0.141 mL/g.

#### 3.3.3. Spectroscopy Analysis

The UV–visible spectrum of RTFP-2b (5 mg/mL) in aqueous solution was measured from 200 to 1000 nm using a multifunctional microplate reader (Multiskan GO, Thermo Fisher Scientific).

FT-IR spectroscopy of RTFP-2b was performed with a Nicolet iZ-10 spectrometer (Thermo Nicolet, Madison, WI, USA). Samples were prepared by mixing RTFP-2b with KBr powder and compressing the mixture into 1 mm pellets for analysis from 4000 cm^−1^ to 400 cm^−1^.

#### 3.3.4. X-Ray Diffraction (XRD) Analysis

The crystallinity of RTFP-2b was characterized at room temperature using an X’Pert Pro X-ray diffractometer (XRD) (PANalytical, Almelo, The Netherlands) with Cu-Kα radiation (λ = 0.15406 nm). Measurements were performed at 40 kV and 40 mA over a 2θ angle range from 5° to 60° with a step size of 0.02° and a scanning velocity of 4°/min.

#### 3.3.5. Micromorphology Structure Analysis

The molecular morphology of RTFP-2b was analyzed using a scanning electron microscope (SEM) (Merlin Compact, Carl Zeiss, Jena, Germany). After sputter-coating with gold, the dried powder sample was mounted on the substrate and imaged at 1.0 kV under high vacuum.

#### 3.3.6. Methylation Analysis

Glycosidic linkage analysis of RTFP-2b was performed using a methylation method based on previous reports [47,60,64] with slight modifications. Briefly, carboxyl groups of uronic acids were initially reduced using CMEC and NaBD_4_/NaBH_4_. The resulting product was permethylated, followed by hydrolysis, reduction with NaBD_4_, and acetylation to yield partially methylated alditol acetates (PMAAs). The PMAAs were analyzed by GC–MS (6890A-5975C, Agilent, Palo Alto, CA, USA) equipped with a BPX70 column (30 m × 0.25 mm × 0.25 μm). High-purity helium was used as the carrier gas at a split proportion of 10:1 with an injection volume of 1 μL. The mass spectrometry conditions were as follows: starting temperature of 140 °C, held for 2.0 min, increased to 230 °C at 3 °C/min, held for 3 min. Data were acquired in SCAN mode across 50–350 *m*/*z*.

#### 3.3.7. Nuclear Magnetic Resonance Analysis

A 30 mg sample of RTFP-2b was digested in 0.5 mL D_2_O to a final concentration of 40 mg/mL. The NMR spectra, including 1D and 2D spectra (1H-NMR, 13C-NMR, COSY, NOESY, HMBC and HSQC), were acquired at 25 °C using a Bruker AVANCE NEO 500M spectrometer system (Bruker, Billerica, MA, USA) running at 500 MHz by Sanshu Biotech. Co., Ltd. (Shanghai, China).

### 3.4. Immunomodulatory Analysis

#### 3.4.1. Cell Culture and Viability Assay

The RAW264.7 cells were obtained from Wuhan Pricella Biotechnology Co., Ltd (Wuhan, China) and incubated in 90% DMEM medium supplemented with 1% dual antibiotics (penicillin and streptomycin) and 10% FBS at 37 °C in a 5% CO_2_ humidified atmosphere. The cells in logarithmic growth phase were collected for further analysis.

The CCK-8 method was used to determine the effect of RTFP-2b on the activity of RAW264.7 cells stimulated by LPS [65]. RAW264.7 cells were added to 96-well plates at a concentration of 5 × 10^5^ cells/mL (100 μL/well) and incubated overnight. Then, RTFP-2b was dissolved in DMEM basal medium and applied to the cells at final concentrations of 100, 200, 300, and 400 μg/mL. After a 1 h incubation, the cells were stimulated with 1 μg/mL LPS solution. Untreated cells were used as blank controls, and cells treated with 1 μg/mL LPS without RTFP-2b served as positive controls. Each group had three replicates. The cells were incubated for 24 h after administration. Then, 10 μL CCK-8 reagent was added to each well, the cells were incubated for another 2 h, and then the absorbance at 450 nm was determined.

#### 3.4.2. Cell Phagocytic Activity

The phagocytic activity of cells was gauged by neutral red assay as described elsewhere with minor modifications [66]. Cells were treated as described in Section 3.4.1 to obtain the blank group, positive control group, and experimental group. After incubation for another 24 h, the supernatant was removed. The cells were rinsed twice with phosphate-buffered saline (PBS), and then 100 μL 0.075% neutral red solution was added to each well. The cells were incubated for 4 h, then the supernatant was removed. After rinsing the cells twice with PBS, 100 μL cell lysis buffer (glacial acetic acid: absolute ethanol, 1:1) was added. The plates were incubated at room temperature for 1 h, and then the absorbance at 540 nm was determined.

#### 3.4.3. Assay of NO and Cytokine Production

According to the cell viability assay in Section 3.4.1, RAW264.7 cells were added to 96-well plates at a concentration of 5 × 10^5^ cells/mL (100 μL/well) for 24 h. The cells were then treated with RTFP-2b at final concentrations of 100, 200, 300, and 400 μg/mL for 1 h, followed by co-treatment with 1 μg/mL LPS for 24 h. The blank control and positive control were the same as above. After the incubation, the supernatant was collected and 50 μL of supernatant was mixed with an equal volume of Griess reagent at room temperature for 10 min, the NO content was then determined according to the instructions of the NO kit at 540 nm with a microplate reader. The levels of TNF-α, IL-6, and IL-1β in the supernatants were measured using ELISA kits according to the manufacturer’s instructions. Briefly, 100 μL of supernatant or standard was added to pre-coated 96-well plates and incubated at 37 °C for 90 min. Following a wash step, a biotinylated detection antibody was added and incubated for 60 min, after which horseradish peroxidase-conjugated streptavidin was applied for a 30 min incubation. The substrate solution was then added, the reaction was terminated with stop solution, and the absorbance was measured at 450 nm.

### 3.5. Western Blot Analysis

Cells were treated as described in Section 3.4.1 to obtain the blank group, positive control group, and experimental group. The supernatant was collected into a sterile centrifuge tube for further analysis. After cell lysis and centrifugation, the protein concentration in the supernatant was determined using the BCA assay, and then a 30 μg protein sample was separated by sodium dodecyl sulfate polyacrylamide gel electrophoresis. The separated proteins were electrophoretically transferred to PVDF membranes under wet conditions. Membranes were incubated with primary and secondary antibodies after blocking, followed by chemiluminescent detection. Band visualization and quantification were performed using a gel imaging system. The relative protein levels of TLR4, NF-kB, p-p65/p65, and p-IkBα/IkBα were calculated and normalized to GAPDH using grayscale intensity ratios.

### 3.6. Statistical Analysis

The results are expressed as the mean ± standard deviation of three determinations. Data were analyzed by one-way ANOVA with SPSS v.23 (IBM, Armonk, NY, USA). Differences were considered statistically significant at *p* < 0.05.

## 4. Conclusions

This study isolated and characterized a novel acidic heteropolysaccharide (RTFP-2b) from *R. tomentosa* fruit. Detailed analyses revealed that the polysaccharide has a molecular weight of 22.995 kDa, and consists of Fuc, Rha, Ara, Gal, Glc, Xyl, Man, Gal-UA, and Glc-UA in a mole ratio of 0.59:9.37:38.68:21.86:3.5:5.28:2.66:14.83:3.22. Structural analysis indicated that RTFP-2b consists of α-L-Ara*f*-(1→, →4)-α-D-Gal*p*A-(1→, →4)-α-D-Gal*p*-(1→, →5)-α-L-Ara*f*-(1→, →3)-α-L-Ara*f*-(1→, →2)-α-L-Rha*p*-(1→, and →3,4,6)-β-D-Gal*p*-(1→. In LPS-stimulated RAW264.7 cells, RTFP-2b demonstrated dose-dependent immunomodulation, with lower concentrations (100–200 μg/mL) promoting phagocytosis and IL-1β production, whereas higher concentrations (300–400 μg/mL) inhibited the NF-κB pathway, though cytokine expression was differentially affected. The galacturonic acid-rich backbone and arabinogalactan branches of RTFP-2b underlie its context-specific bioactivity. Further research is required to evaluate its in vivo efficacy, identify molecular targets, and assess bioavailability for its potential application in functional foods.

## Figures and Tables

**Table 1:** Monosaccharide composition of RTFP-2b.

Monosaccharide	Retention Times of Standards (min)	Retention Times of RTFP-2b (min)	Composition (Molar Ratio/%)
Fuc	4.4167	4.542	0.59
Rha	8.3667	8.584	9.37
Ara	8.8583	9.100	38.68
Gal	11.0917	11.392	21.86
Glc	12.5917	12.984	3.50
Xyl	14.8750	15.400	5.28
Man	15.3917	15.950	2.66
Fru	18.1417	ND	ND
Rib	20.4583	ND	ND
Gal-UA	35.1750	34.017	14.83
Gul-UA	35.8250	ND	ND
Glc-UA	37.3500	36.109	3.22
Man-UA	38.9583	ND	ND

ND = not detected.

**Table 2:** Linkage patterns of RTFP-2b as determined by methylation analysis.

RT (min)	Type of Linkage	Molar Ratio (%)	PMAAs	Mass Fragments (*m*/*z*)
5.847	Rha*p*-(1→	2.80	1,5-di-O-acetyl-6-deoxy-2,3,4-tri-O-methyl rhamnitol	59, 72, 89, 102, 115, 118, 131, 145, 162, 175
6.152	Ara*f*-(1→	20.89	1,4-di-O-acetyl-2,3,5-tri-O-methyl arabinitol	71, 87, 102, 118, 129, 145, 161
8.922	→2)-Rha*p*-(1→	4.04	1,2,5-tri-O-acetyl-6-deoxy-3,4-di-O-methyl rhamnitol	89, 100, 115, 130, 131, 175, 190
9.066	Glc*p*-(1→	2.65	1,5-di-O-acetyl-2,3,4,6-tetra-O-methyl glucitol	87, 102, 118, 129, 145, 161, 162, 205
9.618	→3)-Ara*f*-(1→	6.47	1,3,4-tri-O-acetyl-2,5-di-O-methyl arabinitol	87, 99, 113, 118, 129, 201, 233
10.074	Gal*p*-(1→	6.76	1,5-di-O-acetyl-2,3,4,6-tetra-O-methyl galactitol	87, 102, 118, 129, 145, 161, 162, 205
10.803	→5)-Ara*f*-(1→	9.99	1,4,5-tri-O-acetyl-2,3-di-O-methyl arabinitol	87, 102, 118, 129, 162, 189
11.653	→4)- Xyl*p*-(1→	2.76	1,4,5-tri-O-acetyl-2,3-di-O-methyl xylitol	87, 102, 118, 129, 162, 189
12.529	→2)-Man*p*-(1→	2.45	1,2,5-tri-O-acetyl-3,4,6-tri-O-methyl mannitol	88, 101, 129, 130, 161, 190, 205
12.988	→3)-Gal*p*-(1→	2.77	1,3,5-tri-O-acetyl-2,4,6-tri-O-methyl galactitol	87, 101, 118, 129, 161, 202, 234
13.832	→4)-Gal*p*-(1→	10.26	1,4,5-tri-O-acetyl-2,3,6-tri-O-methyl galactitol	87, 99, 102, 115, 118, 131, 162, 175, 235
13.929	→4)-Gal*p*A-(1→	16.66	1,4,5-tri-O-acetyl-2,3,6-tri-O-methyl galactitol	87, 102, 113, 118, 129, 162, 233
14.496	→2,5)-Ara*f*-(1→	1.24	1,2,4,5-tetra-O-acetyl-3-O-methyl arabinitol	87, 88, 129, 130, 189, 190
15.626	→6)-Gal*p*-(1→	1.65	1,5,6-tri-O-acetyl-2,3,4-tri-O-methyl galactitol	87, 99, 102, 118, 129, 162, 189, 233
15.884	→3,4)-Glc*p*A-(1→	2.23	1,3,4,5-tetra-O-acetyl-2,6-di-O-methyl glucitol	87, 118, 129, 143, 185, 203, 305
17.090	→2,4)-Glc*p*-(1→	1.25	1,2,4,5-tetra-O-acetyl-3,6-di-O-methyl glucitol	87, 88, 99, 113, 130, 190, 233
18.881	→4,6)-Glc*p*-(1→	2.64	1,4,5,6-tetra-O-acetyl-2,3-di-O-methyl glucitol	85, 102, 118, 127, 159, 162, 201, 261
20.514	→3,4,6)-Gal*p*-(1→	2.52	1,3,4,5,6-penta-O-acetyl-2-O-methyl galactitol	97, 118, 129, 139, 160, 333

**Table 3:** ^1^H and ^13^C NMR chemical shifts in RTFP-2b.

Code	Glycosyl Residues	Chemical Shifts (ppm)						
		H1/C1	H2/C2	H3/C3	H4/C4	H5/C5	H6/C6	O-CH_3_
A	α-L-Ara*f*-(1→	5.02	4.06	4.34	4.03	3.65		
		107.5	81.32	77.8	84.02	61.13		
B	→4)-α-D-Gal*p*A-(1→	4.99	3.69	3.46	3.7	3.58		3.58
		98.95	70	71.07	76.31	70	175.44	60.82
C	→4)-α-D-Gal*p*-(1→	5.02	3.73	3.85	4.06	3.98	3.73, 3.64	
		101.63	70.3	73.62	76.38	74.3	63.01	
D	→5)-α-L-Ara*f*-(1→	5.08	4.08	4.22	4.14	3.78, 3.83		
		107.07	83.75	79.11	82.04	66.86		
E	→3)-α-L-Ara*f*-(1→	5.17	4.12	4.31	4.24	3.83		
		109.22	81.65	79.11	84.98	61.1		
F	→2)-α-L-Rha*p*-(1→	5.11	3.93	4.04	3.91	3.7	1.18	
		106.93	83.86	70.27	68.9	70.23	16.57	
G	→3,4,6)-β-D-Gal*p*-(1→	4.43	3.69	3.88	3.63	3.78	3.69	
		103.59	72.84	76.54	75.04	70.75	68.21	

## Data Availability

All data presented are available in this manuscript/Supplementary Materials. Further inquiries can be directed to the corresponding author.

## Supplementary Materials

The following supporting information can be downloaded at: https://www.mdpi.com/article/10.3390/molecules31081365/s1, Figure S1: UV–Vis spectrum of RTFP-2b; Figure S2: XRD patterns of RTFP-2b; Figure S3. Total Ion Chromatogram (TIC) of the methylated derivatives from RTFP-2b. A: (H-); B: (D-); Figure S4. MS/MS spectra of the characteristic peaks (D-; H-); Figure S5. Raw images of Western Blot; Table S1: The molecular parameters of RTFP-2b determined by SEC-MALLS-RI.
