# Supplementary material for: Structural Characterization and Immunomodulatory Activity of an Acidic Polysaccharide from Rhodomyrtus tomentosa (Aiton) Hassk. Fruits
Source: Molecules. 2026 Apr 21;31(8):1365. doi: 10.3390/molecules31081365 (PMC13118346; doi:10.3390/molecules31081365)
Supplement: Supplementary file 1 [file molecules-31-01365-s001.zip › molecules-4200701-supplementary.pdf]

Structural characterization and immunomodulatory activity of an acidic polysaccharide from *Rhodomyrtus tomentosa* (Ait.) Hassk. Fruits

Huihui Yin, Guoqing Yan, Yunfu Huang, Xueyan Zeng, Shenhong He, Tianyan Lan, Wei Liu*

Guangxi Key Laboratory of Veterinary Biotechnology, Key Laboratory of China (Guangxi)-ASEAN Cross-border Animal Disease Prevention and Control, Ministry of Agriculture and Rural Affairs of China, Guangxi Veterinary Research Institute, Nanning, China

*Corresponding Author:

Wei Liu

Tel.: +86 7713395570.

E-mail address: weiliu_0771@163.com

Supplementary Data

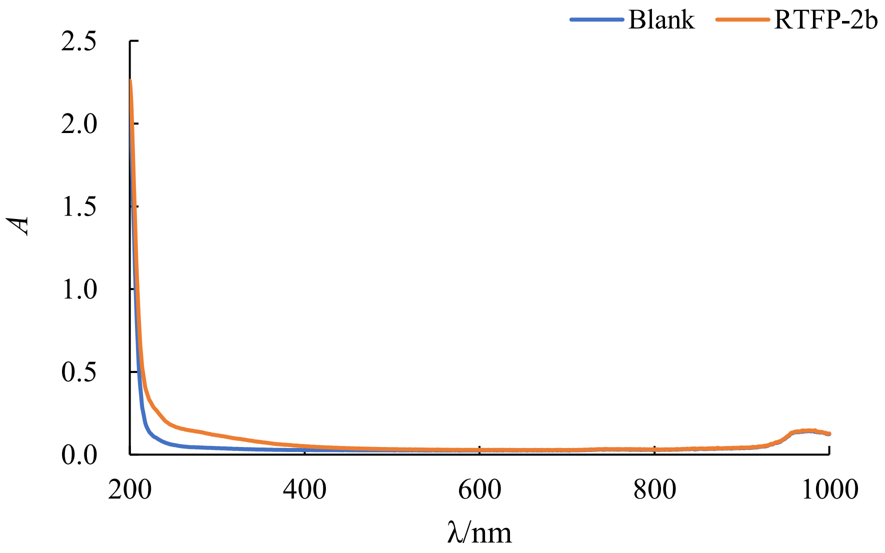

Figure S1. UV–Vis spectrum of RTFP-2b.

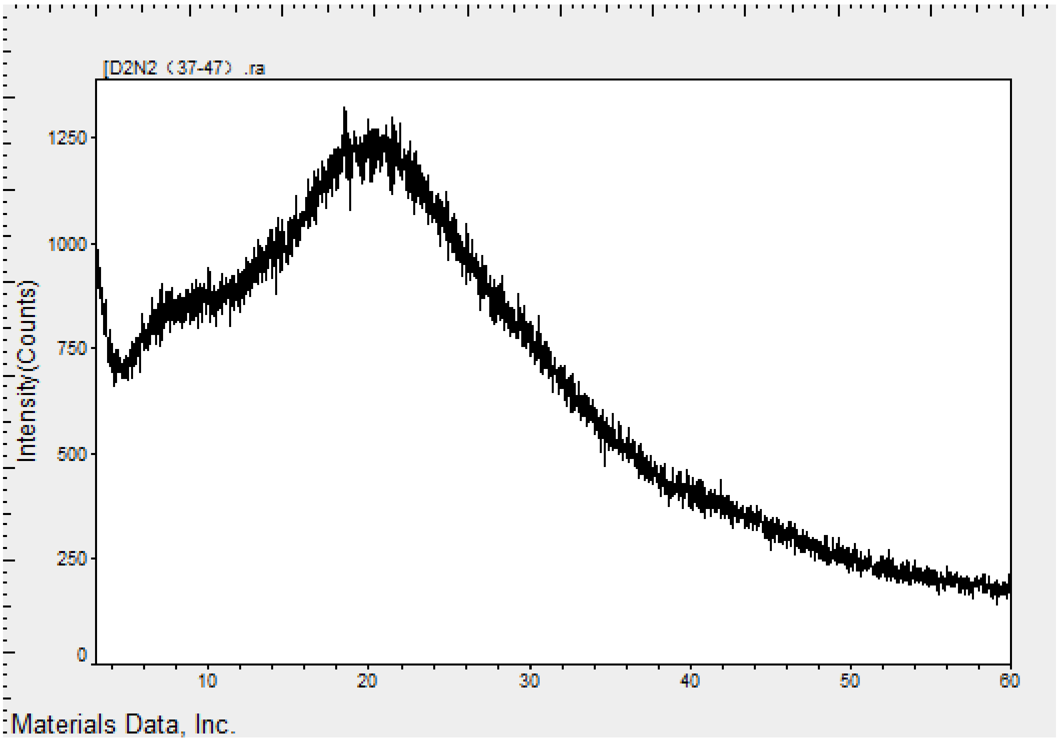

Figure S2. XRD patterns of RTFP-2b.

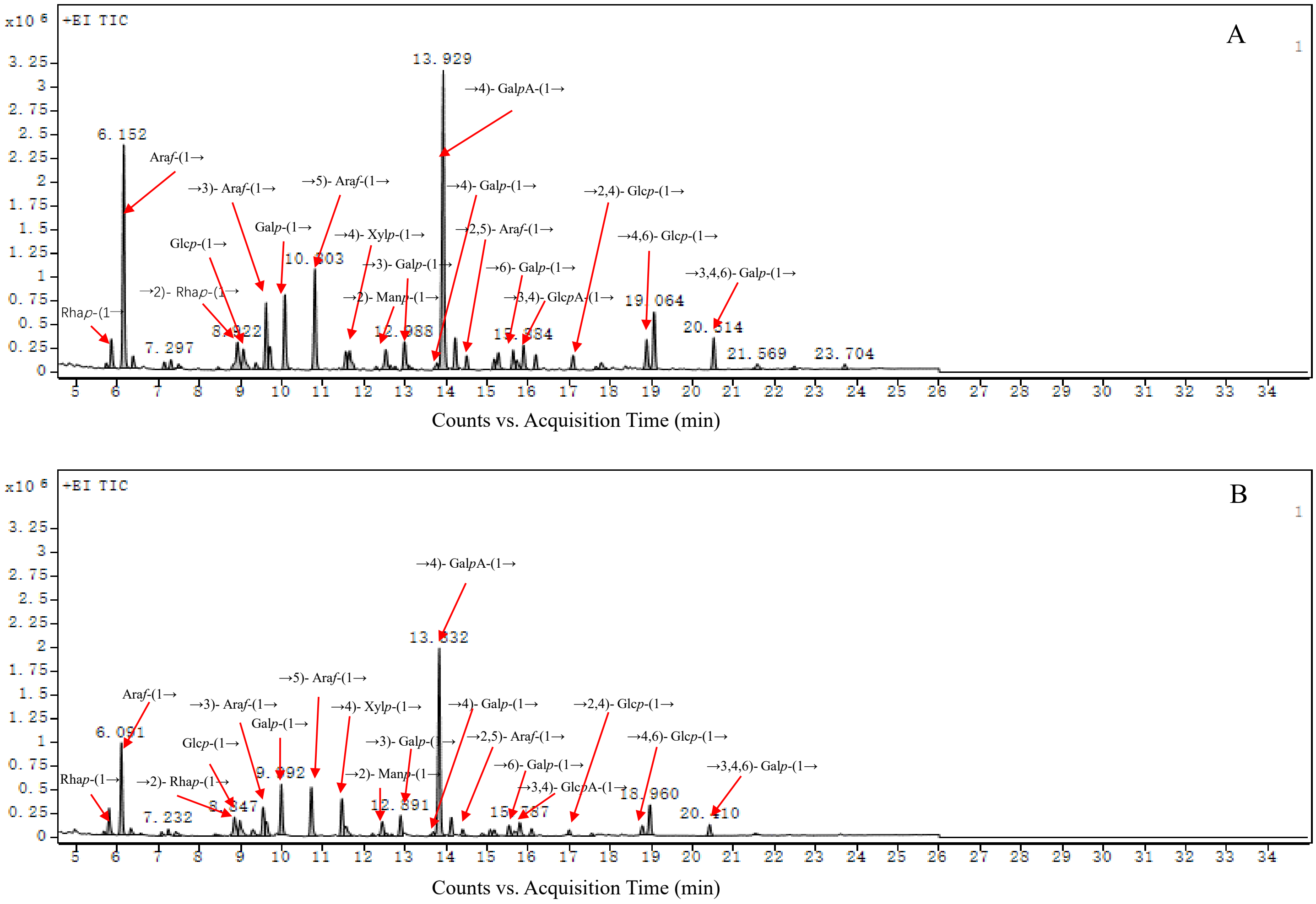

Figure S3. Total Ion Chromatogram (TIC) of the methylated derivatives from RTFP-2b. A: (H-); B: (D-).

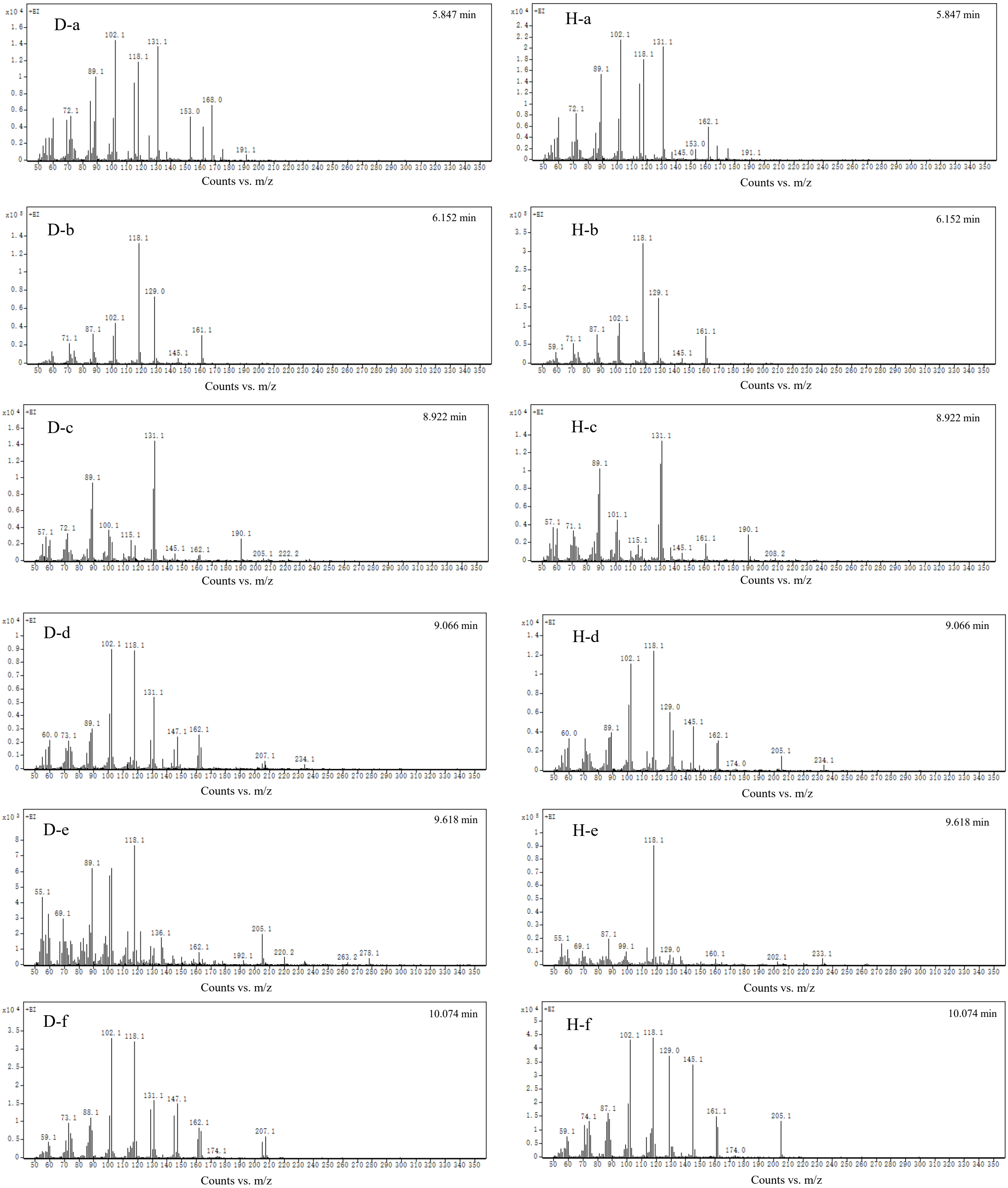
D-a
5.847 min
72.1
89.1
102.1
118.1
131.1
153.0
168.0
191.1
H-a
5.847 min
72.1
89.1
102.1
118.1
131.1
145.0
153.0
162.1
191.1
D-b
6.152 min
71.1
87.1
102.1
118.1
129.0
145.1
161.1
H-b
6.152 min
59.1
71.1
87.1
102.1
118.1
129.1
145.1
161.1
D-c
8.922 min
57.1
72.1
89.1
100.1
115.1
131.1
145.1
162.1
190.1
205.1
222.2
H-c
8.922 min
57.1
71.1
89.1
101.1
115.1
131.1
145.1
161.1
190.1
208.2
D-d
9.066 min
60.0
73.1
89.1
102.1
118.1
131.1
147.1
162.1
207.1
234.1
H-d
9.066 min
60.0
89.1
102.1
118.1
129.0
145.1
162.1
174.0
205.1
234.1
D-e
9.618 min
55.1
69.1
89.1
118.1
136.1
162.1
192.1
205.1
220.2
263.2
278.1
H-e
9.618 min
55.1
69.1
87.1
99.1
118.1
129.0
160.1
202.1
233.1
D-f
10.074 min
59.1
73.1
88.1
102.1
118.1
131.1
147.1
162.1
174.1
207.1
H-f
10.074 min
59.1
74.1
87.1
102.1
118.1
129.0
145.1
161.1
174.0
205.1
Counts vs. m/z

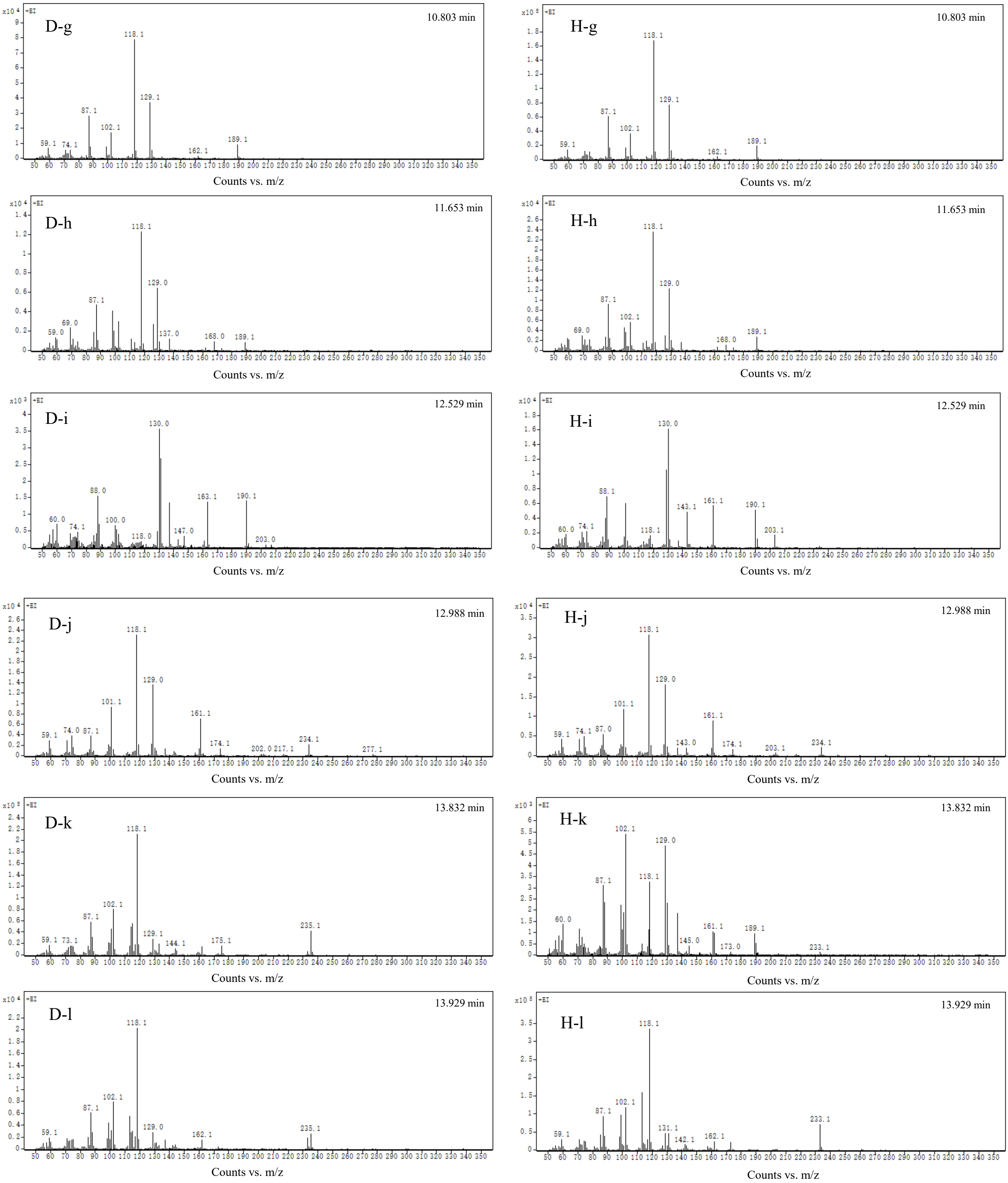

D-g
10.803 min
118.1
129.1
87.1
102.1
59.1
74.1
162.1
189.1
Counts vs. m/z
H-g
10.803 min
118.1
129.1
87.1
102.1
59.1
162.1
189.1
Counts vs. m/z
D-h
11.653 min
118.1
129.0
87.1
69.0
59.0
137.0
168.0
189.1
Counts vs. m/z
H-h
11.653 min
118.1
129.0
87.1
102.1
69.0
168.0
189.1
Counts vs. m/z
D-i
12.529 min
130.0
88.0
163.1
190.1
60.0
74.1
100.0
118.0
147.0
203.0
Counts vs. m/z
H-i
12.529 min
130.0
88.1
143.1
161.1
190.1
60.0
74.1
118.1
203.1
Counts vs. m/z
D-j
12.988 min
118.1
129.0
101.1
161.1
59.1
74.0
87.1
174.1
202.0
217.1
234.1
277.1
Counts vs. m/z
H-j
12.988 min
118.1
129.0
101.1
161.1
59.1
74.1
87.0
143.0
174.1
203.1
234.1
Counts vs. m/z
D-k
13.832 min
118.1
102.1
87.1
59.1
73.1
129.1
144.1
175.1
235.1
Counts vs. m/z
H-k
13.832 min
102.1
129.0
87.1
118.1
60.0
145.0
161.1
173.0
189.1
233.1
Counts vs. m/z
D-l
13.929 min
118.1
102.1
87.1
59.1
129.0
162.1
235.1
Counts vs. m/z
H-l
13.929 min
118.1
87.1
102.1
59.1
131.1
142.1
162.1
233.1
Counts vs. m/z

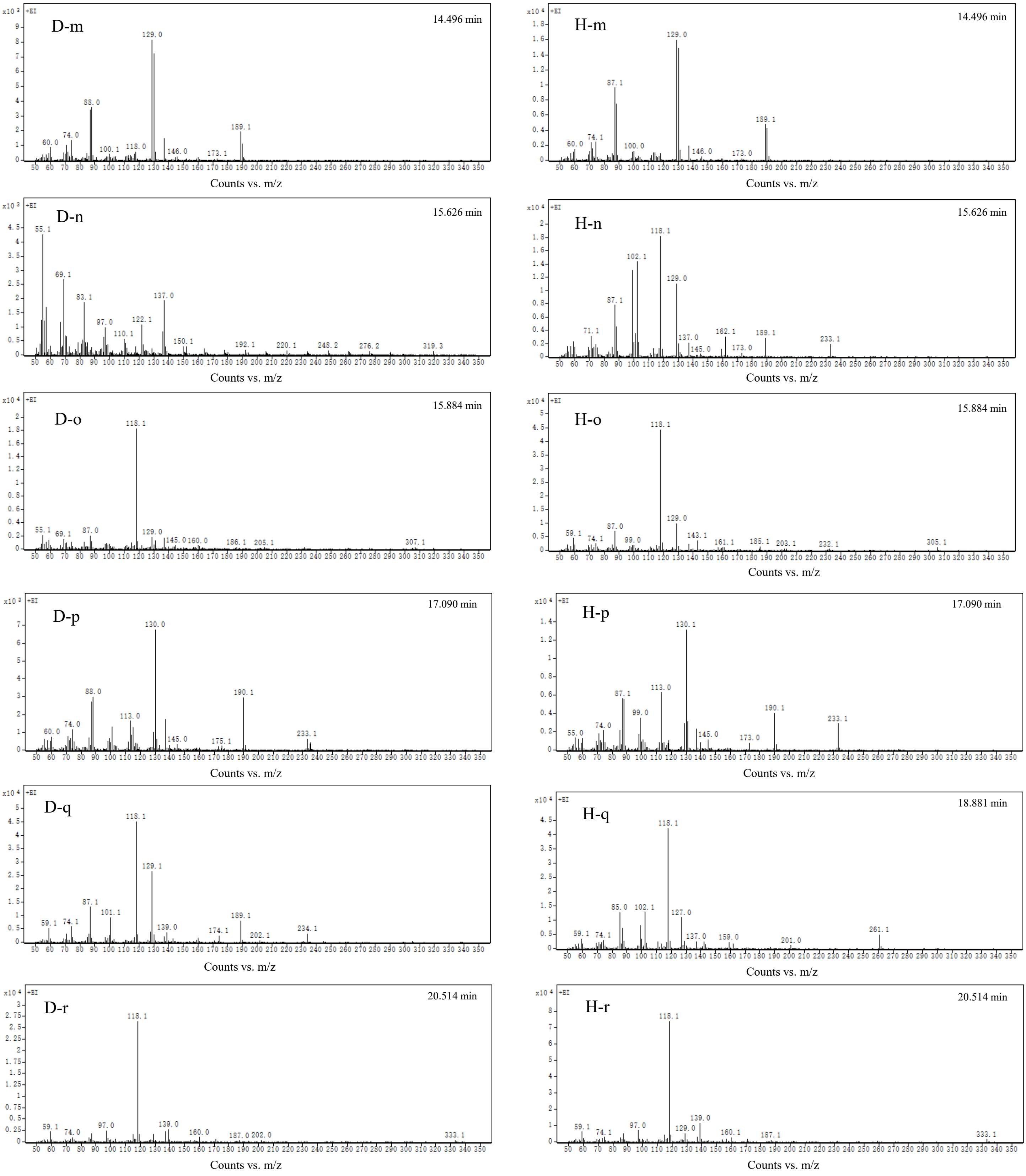

Figure S4. MS/MS spectra of the characteristic peaks (D-; H-).

TLR4

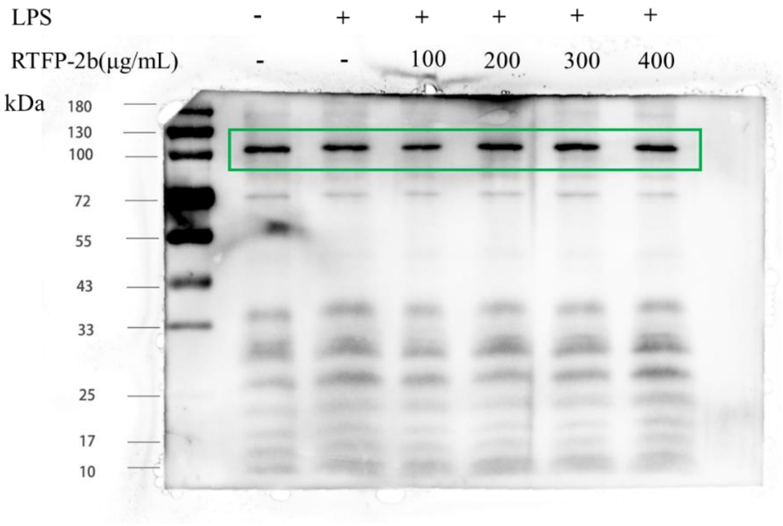

p-IKBα

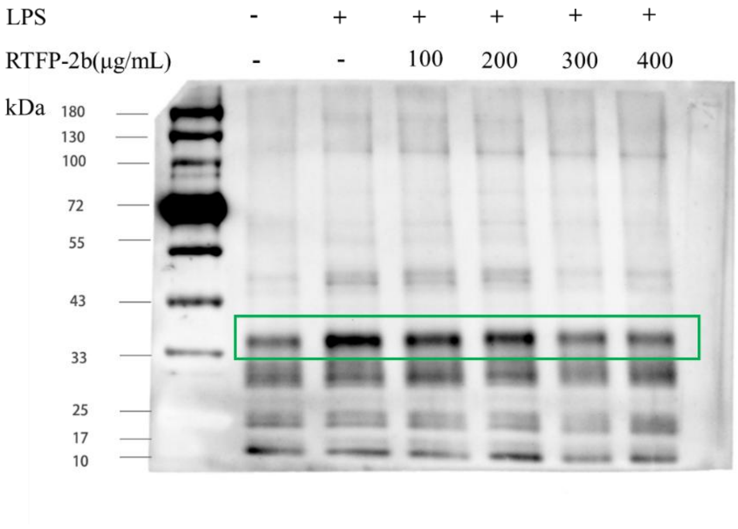

IKBα

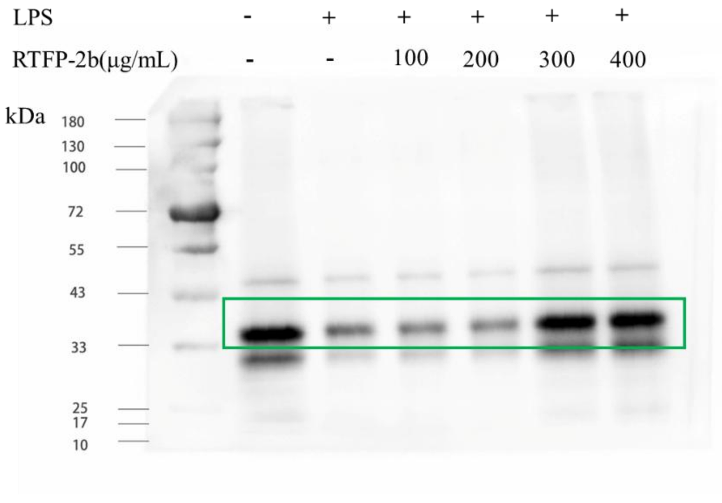

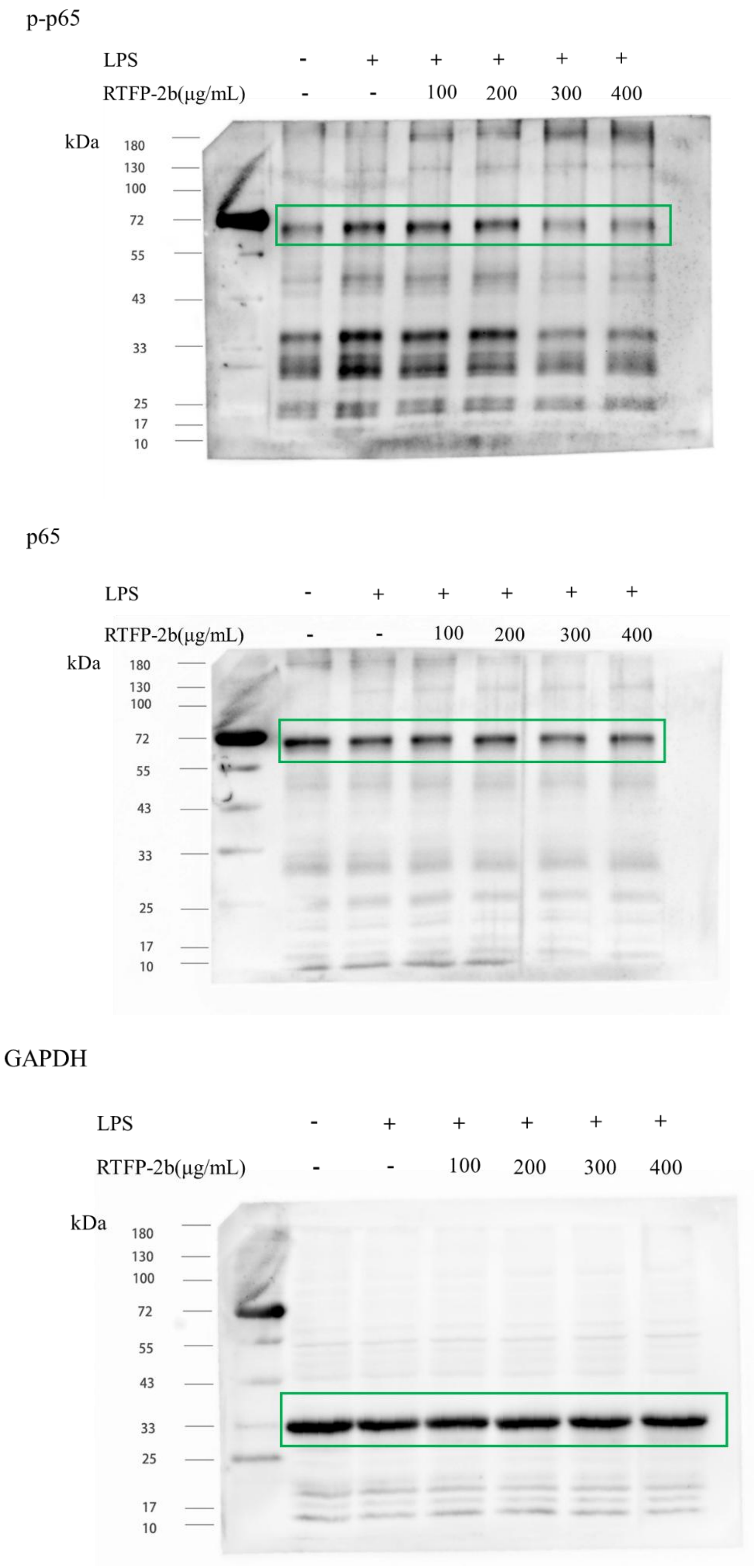

Figure S5. Raw images of Western Blot

Table S1. The molecular parameters of RTFP-2b determined by SEC-MALLS-RI.

Molecular Characteristics	Parameter	Detection Results	Uncertainty
Molar mass moments	*Mn*	16.794 kDa	0.19294
	Mp	10.569 kDa	0.20813
	Mw	22.995 kDa	0.16287
	Mz	33.047 kDa	0.33379
	Polydispersity *(Mw/Mn)*	1.369	0.2525
